# Multilevel Tunnelling Systems and Fractal Clustering in the Low-Temperature Mixed Alkali-Silicate Glasses

**DOI:** 10.1155/2013/263742

**Published:** 2013-06-19

**Authors:** Giancarlo Jug, Maksym Paliienko

**Affiliations:** ^1^Dipartimento di Fisica e Matematica, Università dell'Insubria, Via Valleggio 11, 22100 Como, Italy; ^2^INFN—Sezione di Pavia and IPCF—Sezione di Roma, Italy

## Abstract

The thermal and dielectric anomalies of window-type glasses at low temperatures (*T* < 1 K) are rather successfully explained by the two-level systems (2LS) standard tunneling model (STM). However, the magnetic effects discovered in the multisilicate glasses in recent times, magnetic effects in the organic glasses, and also some older data from mixed (SiO_2_)_1−*x*_(K_2_O)_*x*_ and (SiO_2_)_1−*x*_(Na_2_O)_*x*_ glasses indicate the need for a suitable extension of the 2LS-STM. We show that—not only for the magnetic effects, but also for the mixed glasses in the absence of a field—the right extension of the 2LS-STM is provided by the (anomalous) multilevel tunnelling systems (ATS) proposed by one of us for multicomponent amorphous solids. Though a secondary type of TS, different from the standard 2LS, was invoked long ago already, we clarify their physical origin and mathematical description and show that their contribution considerably improves the agreement with the experimental data. In spite of dealing with low-temperature properties, our work impinges on the structure and statistical physics of glasses at all temperatures.

## 1. Introduction

Glasses are ubiquitous materials of considerable importance for many practical applications; however, for physicists the nature of the glass transition and the ultimate microscopic structure of glasses determining their physical properties remain to this day issues of considerable intellectual challenge [1]. Glasses are normally regarded as fully homogeneously disordered amorphous systems, much alike liquids except for the glassy arrested dynamics close and below the glass transition temperature *T*_*g*_, which leads to an increase of several orders of magnitude in the viscosity for *T* → *T*_*g*_^+^. Nevertheless, this homogeneity is most probably only a useful idealization, for real glasses must always contain some small (in ceramic glasses not so small) concentration of tiny, ordered, or nearly ordered regions of variable size with their own frozen dynamics. Indeed the thermodynamically stable phase of an undercooled liquid would be the perfect crystal; thus, every substance in approaching the crystallization temperature *T*_*c*_ (*T*_*c*_ > *T*_*g*_) from above would spontaneously generate local regions of enhanced regularity (RER) much like a system (a vapour or a paramagnet) approaching its critical temperature is known to develop regions (droplets) resembling the ordered low-temperature phase. These RER are of course to be distinguished from the concept of short-ranged atomic order which is typical of ideal glasses and is restricted to the first few atomic spacings. We are considering in this paper realistic glasses in which a degree of *devitrification* has occurred. The size and concentration of these RER will depend, for example, on the rapidity of the quench leading to the formation of the glass, but also on the chemical composition of the substance, the presence of impurities, and so on. However, on general grounds, even the purest of glasses should contain RER in non-zero concentration and size.

This case has been demonstrated recently for the structure of the metallic glass Zr_50_Cu_45_Al_5_ [2], where a combination of fluctuation electron spectroscopy (FEM) and Monte Carlo simulation (MC) has revealed the presence of crystalline regions of subnanometer size embedded in an otherwise homogeneously amorphous mass of the same composition. It is believed that other metallic glasses should present similar structural features and thus—on general grounds—one would expect that nonmetallic window glasses too, like pure SiO_2_ and all the more so the commercial multisilicates of complex chemical composition, should present a multiphased structure with the size and concentration of the near-crystalline regions, or RER, depending, for example, on composition, quench rate, and the presence of impurities acting as nucleation centres for the RER. Indeed, materials of the general composition (MgO)_*x*_(Al_2_O_3_)_*y*_(SiO_2_)_1−*x*−*y*_ (MAS, in short) are termed *ceramic glasses* (one of the best known commercial examples being Schott's Ceran where Li_2_O replaces MgO, and of course CaO or BaO can also replace or be added to MgO and still yield a ceramic glass). These materials are known to contain microcrystals embedded in an otherwise homogeneously amorphous matrix [3]. This is not surprising, for materials made up of a good glass-former (e.g., SiO_2_, Al_2_O_3_, etc.) and good crystal-formers (e.g., BaO, K_2_O,…) are known to be multiphased [4] with the good crystal-formers generating their own pockets and channels carved out within the otherwise homogeneously amorphous network of the good glass-former's ions [5]. Within these pockets and channels, incipient nano- or even microcrystals may form, but the point of view will be taken in this work that on general grounds even the purest, single-component (e.g., As, SiO_2_) glass-former will be rich in RER unless the quench-rate from the melt is so large as to avoid the formation of crystalline regions or RER.

These refined structural details of glasses are evidently hard to reveal in all and especially the near-ideal cases (no good crystal-formers, no impurities added, and rapid quenches) with the available spectroscopic techniques. For example, X-ray spectroscopy does not reveal nano-crystals below the nanometer size. However, at low and very low temperatures—where all said structural features remain basically unaltered—some recent experimental findings might now improve perspectives with what would appear set to become a new spectroscopy tool. Indeed a series of remarkable magnetic effects have recently been discovered in nonmagnetic glasses (multisilicates and organic glasses) [6–13] with, in the opinion of the present authors, a most likely explanation for the new phenomena stemming precisely from the multiphase nature of real glasses and the presence of the RER or microcrystalline regions in their microscopic structure. In turn, when the multiphase theory shall be fully developed, the magnetic effects could represent a valid new spectroscopic tool capable of characterizing micro- or nanocrystals or even incipient crystals and RER in the real glasses. The key to this possible development is some new exciting physics of the cold glasses in the presence (and even in the absence, as shown in the present paper) of a magnetic field. The magnetic effects in the cold glasses could become, eventually, the amorphous counterpart of the de Haas-van Alphen and Shubnikov-de Haas effects in crystalline solids in determining the real structure of amorphous solids.

Systematic research on the low-temperature properties of glasses has been ongoing for more than 40 years, and some significant theoretical and experimental progress has been made in the understanding of the unusual behaviour of glasses and of their low-temperature anomalies [14–16]. This temperature range (*T* < 1 K) is deemed important for the appearance of universal behaviour (independent of composition), as well as for the effects of quantum mechanics in the physics of glasses. However, to make progress in the understanding of the low-temperature physics of glasses, there remains a wide range of important questions that are still open or only partially answered, particularly in the light of some still poorly understood recent, and even older, experiments in cold composite glasses.

It is well known that cold glasses show somewhat universal thermal, acoustic, and dielectric properties which are very different from those of crystalline solids at low temperatures (below 1 K) [17, 18]. The heat capacity *C*_*p*_ of dielectric glasses is much larger and the thermal conductivity *κ* is orders of magnitude lower than the corresponding values found in their crystalline counterparts. *C*_*p*_ depends approximately linearly and *κ* almost quadratically on temperature *T* (in crystals one can observe a cubic dependence for both properties). The dielectric constant (real part) *ϵ*′ and sound velocity at low frequencies display in glasses a universal logarithmic dependence in *T*. These “anomalous” and yet universal thermal, dielectric, and acoustic properties of glasses are well explained (at least for *T* < 1 K) since 1972 when Phillips [19] and also Anderson et al. [20], independently, introduced the tunnelling model (TM), the fundamental postulate of which was the general existence of atoms or small groups of atoms in cold amorphous solids which can tunnel like a single quantum-mechanical particle between two configurations of very similar energy (two-level systems (2LS)). The 2LS-TM is widely used in the investigation of the low-temperature properties of glasses, mostly because of its technical simplicity. In fact, it will be argued in this paper that tunneling takes place in more complicated local potential scenarios (multiwelled potentials) and a situation will be discussed where the use of a number of “states” greater than two is essential. Moreover, new insight will be given on the role of percolation and fractal theory in the TM of multicomponent glasses. We present in this paper the justification and details of the construction of an extended TM that has been successfully employed to explain the unusual properties of the cold glasses in a magnetic field [21], as well as in zero field when systematic changes in the glass' composition are involved [22].

The linear dependence in ln⁡(*T*) of the real part of the dielectric constant *ϵ*′(*T*) makes the cold glasses useful in low-temperature thermometry, and, normally, structural window-type glasses are expected to be isotropic insulators that do not present any remarkable magnetic-field response phenomena (other than a weak response in *C*_*p*_ to the trace paramagnetic impurities). For some multicomponent silicate glass, it has become possible to measure observable, much larger than expected changes in *ϵ*′(*T*, *B*) (*δϵ*′/*ϵ*′ ~ 10^−4^) already in a magnetic field as weak as a few Oe [6, 7]. A typical glass giving such strong response has the composition Al_2_O_3_-BaO-SiO_2_ thus, a MAS ceramic-glass, herewith termed AlBaSiO. The measurements were made on thick sol-gel fabricated films, a fabrication procedure favoring microcrystal formation [4], cooled in a ^3^He-^4^He dilution refrigerator reaching temperatures as low as 6 mK. Magnetic effects have been reported for both the real and imaginary part of *ϵ* at low frequency (*ω* ~ 1 kHz), for the heat capacity *C*_*p*_ (see, e.g., [21]) and for the polarization echo (where changes in the presence of a magnetic field have been the strongest [10–12]) as well. This behavior was confirmed in other multicomponent glasses, like borosilicate optical glass BK7 and commercial Duran [8], and, moreover, similar effects on *ϵ*′(*T*) have been confirmed in studies of the structural glass *a*-SiO_2+*x*_C_*y*_H_*z*_ in the range 50 < *T* < 400 mK and *B* ≤ 3*T* [9]. Although the dielectric magnetocapacitance enhancement is not dramatic (*δϵ*′(*B*)/*ϵ*′ is typically in the 10^−6^–10^−4^ range), the available measurements show that an unusual effect of the magnetic field is indeed present in the above glasses, yet not measurable in ultrapure SiO_2_ (Suprasil W) and cannot be ascribed to spurious agents (The presence of incipient- or microcrystals in real glasses (sometimes called *devitrification*) should not be considered a spurious effect. On the contrary, the magnetic effects should be a way of characterising the two-phase structure of real glasses.) or to trace paramagnetic impurities (always present in silicate glasses, although in <6 ppm concentration in the case of BK7). Polarization-echo experiments in the AlBaSiO, Duran, and BK7 glasses have also shown considerable sensitivity in the response of the echo amplitude to very weak magnetic fields, and the magnetic effects clearly do not scale with the concentration of paramagnetic impurities [8, 10–12]. Striking magnetic effects, the presence of a novel isotope effect, and remarkable oscillations in the dephasing time have also been reported in studies of the polarization echoes in organic glasses (amorphous glycerol) [13]. However, in terms of a detailed theoretical justification for all of the observed magnetic effects (and the lack of an observable magnetic effect in the acoustic response [23], so far) an explanation relying on a single theoretical model for all of the available experimental data is still missing. We believe the two-phase model reproposed in this paper to be the correct generalization of the standard 2LS-TM that is being sought and here we work out its predictions in zero magnetic field, but for different controlled concentrations of glass-forming and crystal-forming components. In this way, we put our approach to a new test.

The essential behavior of the dielectric response of glasses at low temperatures is well known [17, 18]. According to the standard 2LS-TM (STM from now on), the dielectric constant is predicted to vary like −ln⁡⁡*T* due to the constant density of states of the TS. Above a certain temperature *T*_0_(*ω*), relaxational absorption of the TS becomes important, resulting in an increase of the dielectric constant with temperature proportional to +ln⁡⁡*T* according to the STM. This has been checked experimentally for several glasses. The temperature *T*_0_ of the resulting minimum depends on the frequency *ω* and occurs around 50 to 100 mK in measurements at around 1 kHz.

Some more interesting behavior has been shown by some as yet unexplained data from experiments on the mixed (SiO_2_)_1−*x*_(K_2_O)_*x*_ and (SiO_2_)_1−*x*_(Na_2_O)_*x*_ glasses, studied as a function of the concentration *x* of the good crystal-former at low temperatures [24]. The heat capacity *C*_*p*_(*T*) for these glasses is larger than that for pure vitreous silica and the behavior as a function of *T* is very peculiar for different molar concentrations *x* of potassium or sodium oxide and is not explained by the STM. The heat capacity decreases and then increases again with increasing molar concentration *x* of K_2_O. The minimum in the dielectric constant *ϵ*′(*T*) is observed for *T*_0_ near 100 mK as is typical for these glassy solids. The temperature dependence of *ϵ*′, both above and below *T*_0_, shows however a slope in ±ln⁡⁡*T* qualitatively increasing with increasing concentration *x* of K_2_O. One can notice, moreover, that above the minimum *T*_0_ the relaxation part of *ϵ*′ is increasing faster in slope than the resonant part below *T*_0_ for the same *x* [24], a feature completely unexplained thus far. This work is an indication that not only the magnetic and electric fields influence the properties of glasses, but the concentration of chemical species in the composite materials too (a fact not accounted for by the STM). In this paper, we show in detail how the very same approach that explains the magnetic properties in the multisilicates [21] also provides a quantitative explanation for the above-mentioned composition-dependent physical properties. The picture that emerges regarding the nature of the TS in the multicomponent glasses provides a novel and detailed description of the micro- and nanostructure of the glassy state. In turn, the linear dependence of the concentration *x*_ATS_ of anomalous TS (ATS)—that responsible for the magnetic and composition effects in our theory—on *x* fully corroborates the founding assumptions of our approach.

The paper is organised as follows. In Section 2, we present a detailed justification for the two-phase approach and the construction of the two-species TS model for the amorphous solids at low temperatures. In Section 3, we present the detailed predictions of this model for the dielectric constant *ϵ*′(*T*, *x*) as a function of temperature *T* and composition *x* of alkali oxide (good-crystal former) for the mixed glasses and we compare the predictions with the experimental data [24]. In Section 4, we present the detailed predictions of our model for the heat capacity *C*_*p*_(*T*, *x*) for the mixed glasses and we compare the predictions with the available experimental data [24].  Section 5 contains our conclusions about the nature of the TS; namely, we show how the tunneling “particle” must in fact represent a whole cluster of *N* correlated real tunneling ions in the material. Finally, in the appendix, we work out how the effective tunneling parameters of our model are related, via *N*, to more standard microscopic tunneling parameters. A short preliminary account of this work was published in [22].

## 2. Building Up a Suitable Tunneling Model

The traditional picture [17, 18] viewed the TS, present in low concentration (~10^16^ g^−1^) in the material, associated with the nonequivalence of two (or more) bonding-angle configurations per atomic unit in the amorphous solid's atomic structure. Each TS is represented in the standard case by a particle in an asymmetric (one-dimensional (1D)) double-well potential where, at low-*T*, only the ground states of the two constituent single wells are assumed to be relevant. Consequently, only the two lowest-lying double-well states are taken to determine the physics of each single TS. A 2LS simplified picture then applies, and one can describe the low energy Hamiltonian of each independent TS in terms of an equivalent notation with spin-1/2 pseudospin matrices *σ*_*x*_ and *σ*_*z*_ (Pauli matrices), leading to the compact notation *H*_0_^(2)^ = −(1/2)(Δ*σ*_*z*_ + Δ_0_ *σ*_*x*_) for the Hamiltonian of a single 2LS TS. In matrix form (the so-called well- or position-space representation, 〈*i* | *H*_0_^(2)^ | *j*〉, |*i*〉 being the two unequivalent wells, *i* = 1,2 or *i* = *L*, *R*), this then reads:

(1) $$\begin{array}{c} H_{0}^{\left(2\right)}=-\frac{1}{2}\left(\begin{array}{cc} \Delta & \Delta_{0}\\ \Delta_{0} & -\Delta \end{array}\right). \end{array}$$

Here, the phenomenological parameters Δ and Δ_0_ (known as the energy asymmetry and (twice) the tunnelling matrix element, resp.) represent a way of describing the essential low-*T* relevant features of the full, and yet unknown in its details, TS single-particle Hamiltonian in the effective single-well matrix representation. One obtains ${ℰ}_{1,2}=\pm \left(1/2\right)\sqrt{\Delta^{2}+\Delta_{0}^{2}}$ for the two lowest-lying energy levels, and the physics of the glass is then extracted by assuming (initially) the 2LS to be independent entities in the glass are averaging physical quantities over a probability distribution for the parameters Δ,  Δ_0_ of the standard form ($\overset{-}{P}$ being a material-dependent constant):

(2) $$\begin{array}{c} P\left(\Delta ,\Delta_{0}\right)=\frac{\overset{-}{P}}{\Delta_{0}}. \end{array}$$

This distribution reflects the generally accepted opinion that Δ and −ln⁡(Δ_0_/*ħΩ*) (the latter proportional to the double-well potential barrier *V*_0_ divided by the single-well attempt frequency *Ω*, *V*_0_/*ħΩ*) should be rather broadly distributed in a homogeneously disordered solid. This leads to an almost constant density of states (DOS) and the above STM has been employed with considerable success in order to explain a wide range of physical properties (thermal, dielectric (ac and pulsed), acoustic, etc. [17, 18]) of nonmetallic glasses below 1 K.

There are, however, several drawbacks with the STM as thoughtfully pointed out by Leggett and coworkers [25–27]. For a start, the nature of the TS (and of the two wells of a single 2LS) and that of the motion inside a single TS remain to date completely unknown (We remark that recently, thanks also to the efforts towards the explanation of the magnetic effects [21, 28] and from the study of quantum domain-wall excitations in the cold glasses [29], a picture is emerging of a correlated (or coherent) tunneling cluster of some *N* (charged) particles (atoms or molecules) which is being represented, in the TM, by a single fictitious tunneling particle. In this paper, we argue that *N* ~ 200 in agreement with [29].). Much easier is the diagnostic for the nature of 2LS in the case of disordered crystals, such as Li-KCl or KBr-KCN solutions [30] (we shall come back to disordered crystals later). On general grounds, other types of (multilevel) excitations are always possible in glasses and it is not clear why their distribution of parameters should be so similar (and given by (2)) in all of the amorphous solids. Next, the STM has gathered great consensus for the explanation of many experiments at low temperatures, but in its simplest form (1)-(2) it fails to explain sound velocity shift and adsorption data at low-*T* and the origin of the “bump” in *C*_*p*_ (and “plateau” in *κ*) well above *T*_0_ that goes under the name of *boson peak* (see, e.g., the references in [25–27]). Moreover, the STM fails to explain the remarkable universality of the ultrasonic attenuation coefficient *Q* ^−1^ (roughly, independent of every external parameter and glass chemical composition) below 1 K [31]. To resolve these (and other) difficulties with the STM, Leggett and collaborators have proposed a generic model in the context of anharmonic elasticity theory which can account for all of the significant features of glasses below 1 K, including the super universality of *Q* ^−1^ [25–27].

However, it is hard to see how this generic elastic model can be extended to account for the magnetic and composition-dependent effects in glasses, also considering that in the multicomponent (i.e., real, non model) glasses most of the said universality features (e.g., in *C*_*p*_(*T*, *B*) and *ϵ*′(*T*, *B*) [6, 7, 21] or in *C*_*p*_(*T*, *x*) and *ϵ*′(*T*, *x*) [22, 24]) are lost. Therefore, here we adopt the strategy of resuming the TS approach by means of a completely different (and more modern) justification for the TM and then extend the STM to take the presence of a magnetic field into account and to explain composition-dependent features (this work). In a rather general fashion, the TS can be thought of as arising from the shape of the theoretical potential-energy landscape *E*({**r**_*i*_}) of a glass as *T* is lowered well below the glass freezing transition *T*_*g*_. The concept of free-energy landscape was introduced, for example, by Stillinger [32, 33] and successfully employed in the study of glasses (e.g., [1]) and spin-glasses (e.g., [34, 35]). A large number of local and global minima develop in *E*({**r**_*i*_}) as *T* → 0, the lowest-energy minima of interest being made up of *n*_*w*_ = 2,3,… local wells separated by shallow energy barriers. At low-*T*, these configuration-space local multiwelled potentials are our TS, and it seems reasonable to expect that the *n*_*w*_ = 2-welled potentials (2LS) should be ubiquitous in this picture. These should be thought of as an effective representation of local “*tremblements*” of the equilibrium positions {**r**_*i*_^(0)^} of some of the glass atoms/ions' positions spanning over a large number of near-neighbors' distances (unlike in the case of disordered crystals, where the TS are known to be rather well-localized dynamical entities). Hence, just as the *n*_*w*_ = 2-welled case is possible, so ought to be the *n*_*w*_ = 3,4,…-welled situations which would also be local rearrangements involving several atoms/ions/molecules. The concentration of these local potentials should not necessarily decrease exponentially with increasing *n*_*w*_, in glasses, as it is known to happen for the disordered crystals (2LS present with probability *c* ^2^, 3LS with *c* ^3^, 4LS with *c* ^4^,… etc., *c* being the defects' percent concentration).

**Figure 1:**
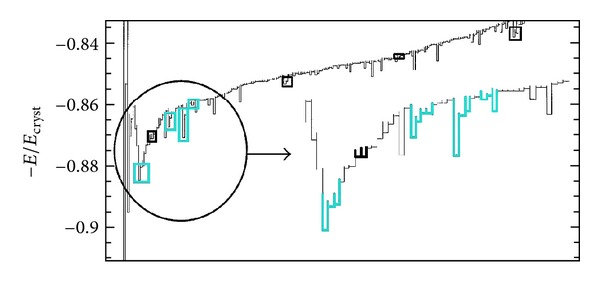
(Color online) The energy landscape (for *ρ* = 1 Lennard-Jones density, adapted from [36]) of a toy glass model, with highlighted multiwelled potentials (black the 2LS, light blue the 3LS, 4LS,…).

We can reason this out over the quantitative description of the glassy energy landscape of a model situation, as was studied by Heuer [36] who considered the molecular-dynamics (MD) simulation data of a toy glass made up of several (13 or 32) particles interacting through a Lennard-Jones potential and with periodic boundary conditions applied. Adopting a suitable 1D projection procedure, where a “distance” between two local total energy minima is (not completely unambiguously) defined, the 1D position of a local minimum is somehow attained and the energy landscape of the model system can be charted out.  Figure 1 reports this chart for the total energy landscape for a given density (from [36]). Beside the deep minimum of the crystalline configuration, a large number of local minima are visualized and then a suitable definition of local double-welled potentials (2LS) is adopted to classify couples of adjacent minima constituting a single tunneling 2LS (highlighted in black, Figure 1). This definition guarantees that at low temperatures a “particle” subjected to any such local potentials will switch between both minima without escaping to a third minimum. Interestingly, the distribution of the tunneling parameters Δ, Δ_0_ (suitably defined) for these 2LS could also be evaluated from MD simulations of the above toy model, and this *P*(Δ, Δ_0_) turned out to be not so perfectly flat as a function of Δ as implied by (2). Rather, an increase (though no divergence) of probability for 2LS with Δ → 0 was measured in previous MD simulations [37]. Still, Figure 1 also allows for tunneling multiwelled local potentials to be identified, and we have highlighted (in light blue) some of them (three- and four-welled local potentials). The requirement that a “particle” subjected to such multiwelled local potentials should not escape (at low-*T*) to foreign minima has been equally respected, and one can see that these multiwelled situations are not at all rare. We therefore believe that 3LS, 4LS, and so on should also be considered in the TM. The reduced Hamiltonians (well- or position-representation) for these local multiwelled potentials can be easily written down, as generalizations of (1). For *n*_*w*_ = 3 (3LS),

(3) $$\begin{array}{c} H_{0}^{\left(3\right)}=\left(\begin{array}{ccc} E_{1} & D_{0} & D_{0}\\ D_{0} & E_{2} & D_{0}\\ D_{0} & D_{0} & E_{3} \end{array}\right), \end{array}$$

where *E*_1_,  *E*_2_,  *E*_3_ are random energy asymmetries between the wells chosen to satisfy ∑_*i*=1_^3^ *E*_*i*_ = 0 and taken from an appropriate probability distribution (see below), together with the tunneling parameter *D*_0_ > 0 (see below). For *n*_*w*_ = 4 (4LS):

(4) $$\begin{array}{c} H_{0}^{\left(4\right)}=\left(\begin{array}{cccc} E_{1} & D_{1} & D_{2} & D_{1}\\ D_{1} & E_{2} & D_{1} & D_{2}\\ D_{2} & D_{1} & E_{3} & D_{1}\\ D_{1} & D_{2} & D_{1} & E_{4} \end{array}\right), \end{array}$$

where *E*_1_,  *E*_2_,  and *E*_3_,  *E*_4_ are random energy asymmetries taken from an appropriate probability distribution, together with the tunneling parameters *D*_1_ (n.n. well hopping) and *D*_2_ (n.n.n. hopping, |*D*_2_ | ≪|*D*_1_|). These are simple, possible choices; clearly, other special-purpose generalizations of the 2LS matrix Hamiltonian are possible and we believe that the 3LS of (3) is the minimal generic multiwelled potential which can take the magnetic field into account [21] (the 2LS Hamiltonian of (1) could also be adjusted for this purpose; however, the energy spectrum would be totally insensitive to *B*). One can easily convince oneself, at this point, that as long as the energy parameters of the above multiwelled effective Hamiltonians obey the usual uniform distribution (see (2), suitably reformulated) as is advocated by the STM, the DOS *g*(*E*) will remain (roughly) a constant. It is then to be expected that all these multiwelled local potentials will give rise to the very same physics as in the *n*_*w*_ = 2 case and that thus, in practice, the 2LS choice represents the appropriate minimal model for all of the extra low-energy excitations characterising amorphous solids at low-*T*. It is clear from the above discussion, however, that the 2LS tunneling “particle” is not atomic particle at all, but, on general grounds, it rather represents the local rearrangements of a good number of real particles (ions or molecules).

**Figure 2:**
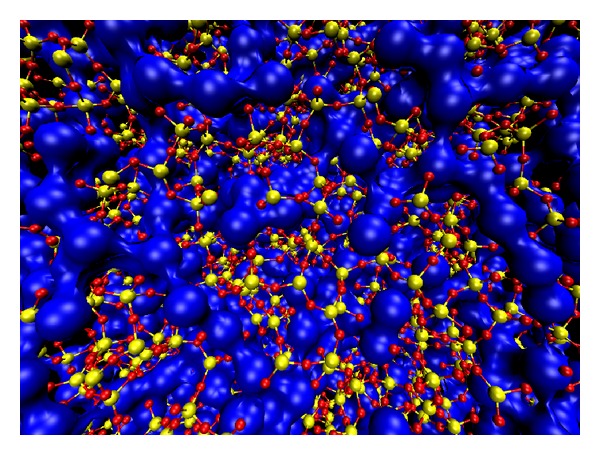
(Color online) Molecular dynamics snapshot of the structure of sodium trisilicate at 2100 K at the density *ρ* = 2.2 g cm^−3^. The big blue spheres that are connected to each other represent the Na atoms. The Si–O network is drawn by yellow (Si) and red (O) spheres that are connected to each other by covalent bonds shown as sticks between Si and O spheres (from [5], by permission).

**Figure 3:**
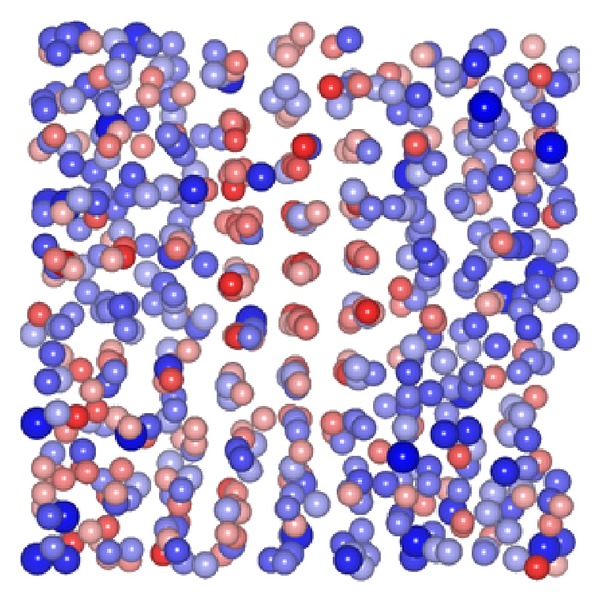
(Color online) A region including the crystal-like supercluster from a snapshot of the model simulation—incorporating fluctuation electron microscopy data—of the Zr_50_Cu_45_Al_5_ metallic glass at 300°C (from [2]). The atomic separation distances of the middle zone are about 0.25 nm. This is a first realistic image of a crystal embryo in a glass; this object should not be confused with the concept of short-range order in ideal glasses.

All changes if the glass is made up of a mixture of network-forming (NF) ions (like those of the good glass-forming SiO_4_ or (AlO_4_)^−^ tetrahedral groups) as well as of network-modifying (NM) ions (like those of the good crystal-forming K^+^ or Na^+^, or Ba^2+^,… from the relative oxides) which, these last ones, could act as nucleating centres for a partial devitrification of the glass, as is known to occur in the multicomponent materials [38–41]. Indeed, the NM-ions of the good crystal-formers are termed “glass modifiers” in the glass chemistry literature [42] since they do not become part of the interconnected random network but carve out their own pockets and channels within the glassy network [5, 43].  Figure 2 (courtesy from Meyer et al. [5]) shows a snapshot of a MD simulation of the glass having composition Na_2_O·3(SiO_2_) (or (Na_2_O)_0.25_ (SiO_2_)_0.75_) at 2100 K (above *T*_*g*_, in fact) in which the nonnetworking NM Na-atoms are put in evidence (big blue spheres). Simulations and experiments in the multisilicates definitely show that the NM-species in part destroy the networking capacity of the NF-ions and form their own clusters inside the NF-network [5]. The chance for these NM-clusters to be the nest of RER, incipient- or actual microcrystals is obviously very good, considering that these clusters are made of good crystal-forming atoms. However, on general grounds and as discussed in Introduction, we shall take the attitude that even the purest single-component glasses will contain RER in some measure.  Figure 3 (from [2]) shows one such RER within a snapshot from a joint MC-simulation/FEM-measurement on the metallic glass Zr_50_Cu_45_Al_5_. The picture clearly shows an embryo crystal which could not grow to macroscopic size due to the arrested dynamics below *T*_*g*_; such structures are expected to ubiquitous in all glasses, metallic and nonmetallic [44], except that they are difficult to observe with the available spectroscopic tools when subnanometric in size. The concentration and size of these RER will dictate whether magnetic- or composition-effects become measurable in the low-*T* experiments. *a*-SiO_2_ in its purest form (Suprasil W) revealed no measurable magnetic effects [6, 7, 9–12].

**Figure 4:**
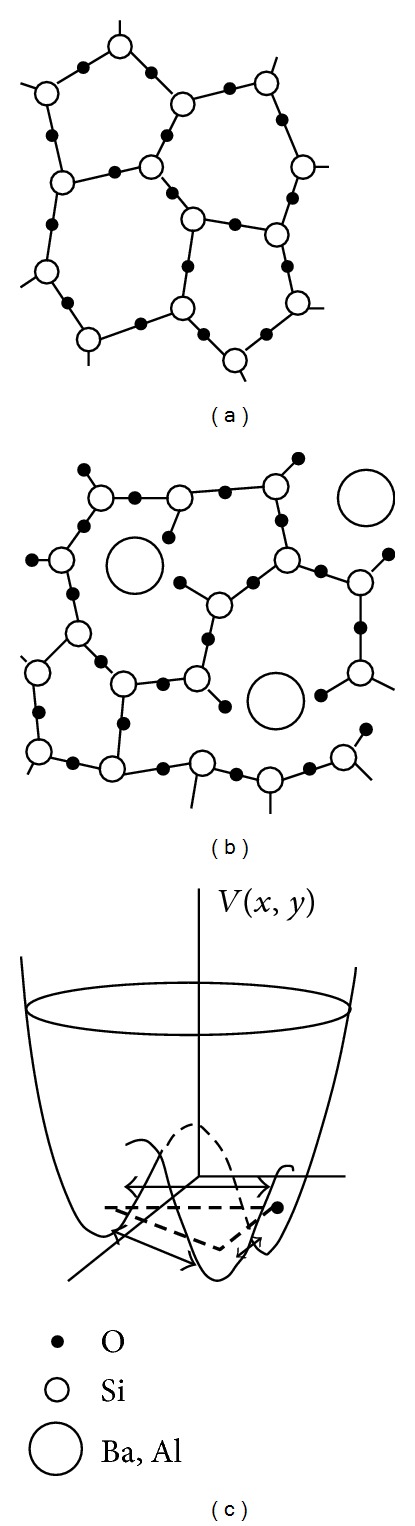
Two-dimensional representation of the plausible source of magnetic-field sensitive (anomalous) tunneling systems in, for example, the AlBaSiO glass. The tight vitreous-SiO_2_ structure (a) is broken up by the Al- and large Ba-atoms (b), thus leaving many metal ions free to move in a *n*_*w*_-minima (soft) tunneling potential, with *n* ≥ 3 (c). The unbroken Si–O–Si bond dynamics, if any, is of the usual 2LS-type.

It goes without saying that TS forming in the proximity and within these RER or microcrystalline regions will require a completely different mathematical description, in particular the possibility of having more than two wells affords a more realistic description of the energy landscape. Hence, *n*_*w*_ > 2 multiwelled systems inside the glass-modifying NM-pockets and -channels should follow some new energy-parameters' distribution form when some degree of devitrification occurs, leading to entirely new physics. One of the present authors has proposed that precisely this situation occurs inside the magnetic-sensitive multicomponent glasses [21], and in this paper we show how this theory explains the *B* = 0 composition-dependent dielectric and heat capacity data of [24] as well. Instead of the standard 1D double-welled (W-shaped) potential, leading to (1), which continues to describe the ordinary tunneling 2LS inherent to the homogeneously disordered *a*-SiO_2_ network, we take for the TS nested in or near the RER, crystal embryos or micro-crystals, the model of a “particle” having charge *q* and moving in a *n*_*w*_-welled 3D potential of the shape displayed, for *n*_*w*_ = 3, in Figure 4 for the 2D (*x*, *y*)-space. The hopping Hamiltonian of a single, non interacting tunneling 3LS has therefore the form (for a fictitious second-quantization particle in the well-coordinate representation)

(5) $$\begin{array}{c} H_{0}^{\left(3\right)}=\sum_{i=1}^{3}E_{i}c_{i}^{†}c_{i}+\sum_{i\neq j}D_{0}c_{i}^{†}c_{j}+\text{hc}., \end{array}$$

and is described in matrix form by (3) (where in fact 〈*i* | *H*_0_^(3)^ | *j*〉 is displayed, |*i*〉 (*i* = 1,2, 3) denoting the single-well ground states). This is our minimal generic model for a multiwelled TS. The parameter *D*_0_ is chosen positive (contrary to custom in the STM, indeed −(1/2)Δ_0_ < 0 in (1)) for a good number of reasons. First, due to the possible softness of the local NM-potential, since indeed in general [17, 18] *D*_0_≃*a* *ħΩ* *e* ^−*bV*_0_/*ħΩ*^, *a* and *b* being numbers such that for *V*_0_ ≳ *ħΩ* *a* > 0 and *b* = *O*(1) can arise [17, 18, 21]. This choice is still compatible with the concept of tunneling and at the same time yields rather large values of *D*_0_ ≈ *ħΩ*. On more general grounds, however, one should take into account that the tunneling “particle” is not moving in a vacuum, but is embedded in a solid that is, for the most part deprived of microscopic dynamics, at low-*T*. Thus, the surrounding frozen atoms are taking a part in the determination of the tunneling particle's lowest stationary states. In the case of a perfectly D_3_-symmetric local *n*_*w*_ = 3 welled potential of the type depicted in Figure 4, Hamiltonian (3) leads to a doubly degenerate ground state and a first excited nondegenerate state (as is easily verified from (3) if *E*_1_ = *E*_2_ = *E*_3_). This may seem unphysical and yet Sussmann has demonstrated, in a remarkable paper [45], that for electrons trapped in a crystal (or equivalently in a glass) the situation above described is realised whenever the trapping potential is multiwelled with a triangular (*n*_*w*_ = 3) or tetrahedral (*n*_*w*_ = 4) well-centers geometry. The binding of the seemingly antibonding ground state is then guaranteed by the TS interaction with the rest of the solid. This reasoning is irrelevant for the STM-2LS parameter Δ_0_, since both positive and negative signs for this parameter yield the same physics. If *n*_*w*_ > 2, the sign will matter and Sussmann's work shows that the choice *D*_0_ > 0 is physically justified for an embedded particle (or vacuum) in the glass. Finally, it will be shown in Conclusions that in fact the tunneling “particle” cannot be considered a single atom, ion, or molecule, but rather it represents a cluster of *N*-correlated tunneling atomic-scale particles, with *N* ≈ 200. Then, it is reasonable to expect that the ground state of such a cluster might be near-degenerate; so our choice *D*_0_ > 0 for the effective single tunneling “particle” is sound and not in conflict with any general quantum-mechanical principle. This *D*_0_ > 0 is the major assumption for the multiwelled TS theory. It should be mentioned, however, that multiwelled potentials appear also in the Jahn-Teller quantum phenomena [46] and that in that context degenerate ground states are also commonplace. In the present situation, however, the disorder inherent in glasses does not allow for a detailed symmetry analysis.

**Figure 5:**
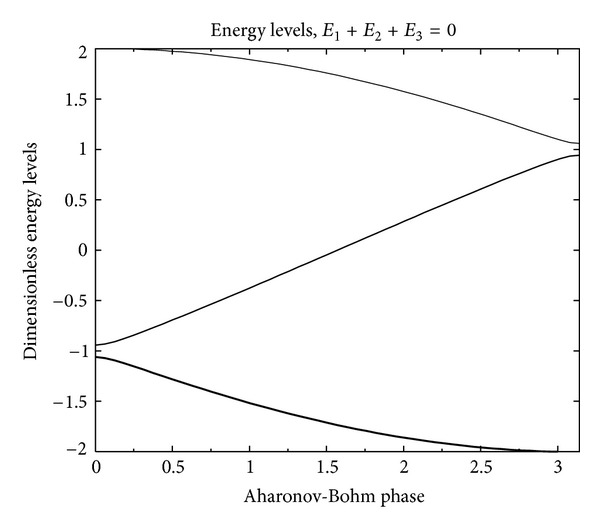
Variation within the magnetic Aharonov-Bohm phase *ϕ* of the energy spectrum (units *D*_0_ = 1) for a choice of *E*_1_, *E*_2_, and  *E*_3_ with *D*/*D*_0_ = 0.01. In this work, we are interested in the *ϕ* = 0 limit of this spectrum, which can be treated, at low-*T*, as that of an effective 2LS.

At this point, we make a choice for the probability distribution of the parameters *E*_1_,  *E*_2_,  *E*_3_, and *D*_0_ of a tunneling 3LS nesting in the proximity of a RER, crystal embryo, or micro-crystal (one could also work with a *n*_*w*_ = 4 model potential; in the appendix we show that essentially the same results can be attained). This is dictated by the fact that near-degeneracy (*E*_1_ = *E*_2_ = *E*_3_) must be favored, yet not fully attained for the wells' energy asymmetries of one such 3LS. We thus choose, assuming again the tunneling potential barriers to be broadly distributed,

(6) $$\begin{array}{c} P_{\text{ATS}}\left(E_{1},E_{2},E_{3};D_{0}\right)=\frac{P^{*}}{\left(E_{1}^{2}+E_{2}^{2}+E_{3}^{2}\right)D_{0}}. \end{array}$$

which has the advantage of making use of a dimensionless material-dependent parameter P*: *P*_ATS_(*E*_1_, *E*_2_, *E*_3_; *D*_0_), multiplied by the concentration *x*_ATS_ of these anomalous (multiwelled, and now near-degenerate) tunneling systems (ATS), is the probability of finding one such ATS per unit volume. In the following, *x*_ATS_ will be absorbed in the parameter P*. This choice for *P*_ATS_ has provided a good description of the experimental data for the multisilicates in a magnetic field [21], when in the Hamiltonian (3) (or equivalently (5)) *D*_0_ at position (*i*, *j*) is replaced with *D*_0_ *e* ^*iϕ*_*ij*_^ (*ϕ*_*ij*_ being the appropriate Peierls phase). As was shown in [21], the spectrum of this *B* > 0 modified 3LS Hamiltonian (3) is formally given by (using Viète's formula for the cubic equation's solutions):

(7) $$\begin{array}{c} \frac{{ℰ}_{k}}{D_{0}}=2\sqrt{1-\frac{\sum_{i\neq j}^{}E_{i}E_{j}}{6D_{0}^{2}}}\cos\left(\frac{1}{3}\theta +\theta_{k}\right)\\ \cos\theta =\left(\cos\phi +\frac{E_{1}E_{2}E_{3}}{2D_{0}^{3}}\right){\left(1-\frac{\sum_{i\neq j}^{}E_{i}E_{j}}{3D_{0}^{2}}\right)}^{-3/2}, \end{array}$$

(with *k* = 0, 1, 2 and *θ*_*k*_ = 0, +(2/3)*π*, −(2/3)*π* distinguishing the three lowest eigenstates) and for a choice of *E*_1_, *E*_2_, *E*_3_ and $D_{0}\gg \sqrt{E_{1}^{2}+E_{2}^{2}+E_{3}^{2}}$ (near-degenerate limit); this is shown in Figure 5. One can see that for very small *ϕ* (the Aharonov-Bohm phase proportional to the magnetic field *B*: *ϕ* = 2*π*Φ(**B**)/Φ_0_, Φ(**B**) = **S**_Δ_ · **B** being the flux through the single ATS (see also the appendix)), the spectrum consists of an isolated near-degenerate doublet which is well separated from the higher excited states. We shall exploit the *ϕ* = 0 limit of this description for an explanation of the composition-dependent experiments.

It should be stressed at this point that in the absence of a magnetic field, like in this work, one could make use of a 2LS minimal model for the description of the ATS, *H*_0_^(2)^(*E*_1_, *E*_2_; *D*_0_), and with the distribution $P(E_{1},E_{2};D_{0})=P^{*}/D_{0}\sqrt{E_{1}^{2}+E_{2}^{2}}$ ensuing from the proximity of RER or incipient microcrystallites. It was shown in [21] that, at least for the heat capacity, this leads to the same physics as obtained from the 3LS multiwelled model. There is no harm in using, for the ATS nesting in the incipient crystalline regions, a more realistic minimal generic multiwelled model like the above 3LS Hamiltonian *H*_0_^(3)^ which better approximates the physical reality of the energy landscape. Moreover, the model for the composition-dependent effects remains the very same used for the magnetic effects, and many results already obtained for that theory can be exploited by setting simply *B* = 0. We remark, also, that a distribution of the type (6) for the energy asymmetry was already proposed for the explanation of low-*T* experiments with mesoscopic Au and Ag wires [47], where TS (of standard 2LS type) were advocated and where the polycrystallinity of metals must be accounted for.

**Figure 6:**
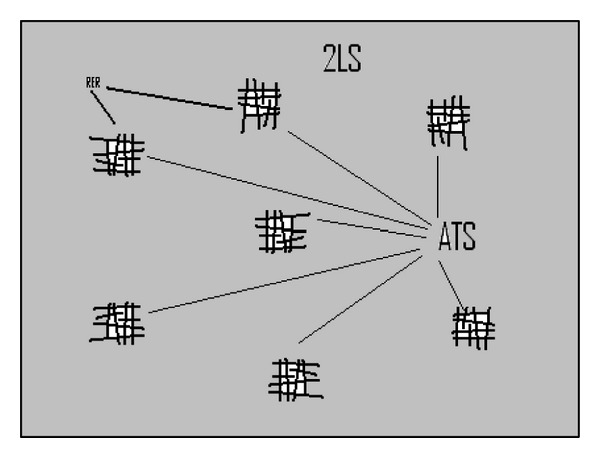
A 2D cartoon of the chocolate-like, ceramic-glass structure of a real glass, in which partial devitrification has occurred, with the location of its low-*T*, two-species TS. In the randomly networked bulk of the material sit the STM-2LS, with their own concentration *x*_2LS_, whilst within and in the proximity of the incipient crystallites nest the ATS, with their own bulk concentration *x*_ATS_, each being described by (3) and (6). We expect *x*_ATS_ < *x*_2LS_ and that *x*_ATS_ → 0 in the best glasses.

In summary, we have fully justified the extended TM which we have used in [21] and which we exploit also in this paper. The realistic glass is recognized to have a structure resembling that of chocolate [48] (or of opals) and as is pictured in the cartoon in Figure 6: a homogeneously-disordered networked solid in which (at low-*T* in the glass) only standard 2LS are present with their own concentration *x*_2LS_ and in which incipient crystallites are embedded (for chocolate, these would be sugar crystals). In the proximity or within these crystallites are nested the ATS, with their own concentration *x*_ATS_ in the solid and with their own quantum mechanics and statistics defined by the minimal generic model represented by (3) and (6). This is by no means an ad hoc model, since the very same model would describe TS in all types of real metallic and nonmetallic glasses and quantitatively explain all of the low-*T* experiments in nonmetallic glasses tackled so far.

## 3. Predictions for the Dielectric Constant

The 2LS-STM has been successful in the semiquantitative explanation of a variety of interesting thermal, dielectric, and acoustic anomalies of structural glasses at temperatures *T*< 1 K [14–18], the physics of cold glasses being important not only for their universalities, but also because of their link with the physics of the glass transition (see, e.g., [49, 50]). Beside the linearity in *T* behavior of the heat capacity *C*_*p*_, it is believed that the linearity in ±ln⁡⁡*T* behavior of the real part of the frequency-dependent dielectric constant *ϵ*′(*T*, *ω*) represents a cogent characterization of the glassy state at low temperatures. We begin by deriving this behavior and putting it to test on data for *ϵ*′ for pure amorphous silica (i.e., no measurable ATS effects).

In the presence of an applied electric field **F**, we must add the dipole energy −**F** · **p**_0_ to the parameter (1/2)Δ in the expression (1) for the low-energy Hamiltonian *H*_0_^(2)^. We can express the permittivity (strictly speaking, the polarization) as *ϵ* = −∂^2^ *f*(*F*)/∂*F* ^2^|_*F*=0_, where *f*(*F*) = −(1/*k*_*B*_ *T*)ln⁡*Z*(*F*) represents the free energy per unit volume. The statistical average implies also an integration over the two parameters of the 2LS, Δ and Δ_0_, according to the distribution given by (2). We can write the partition function in terms of the energy levels *E*_1,2_: *Z* = *e* ^−*ℰ*_1_/*k*_*B*_*T*^ + *e* ^−*ℰ*_2_/*k*_*B*_*T*^.

**Figure 7:**
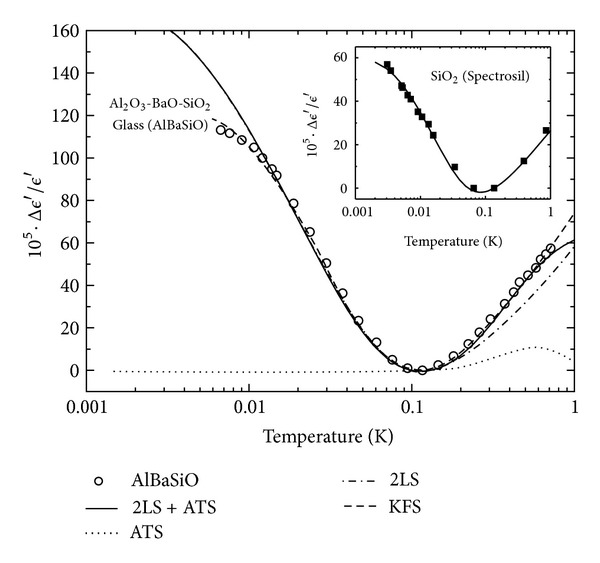
Dielectric signature of pure *a*-SiO_2_ (inset) and AlBaSiO (main) glasses. SiO_2_ data [53], fitted with (8), display a −2 : 1 2LS-TM behavior. AlBaSiO data [54] display rather a −1 : 1 behavior, yet could be fitted with (8) (dashed line) [54] with a large Δ_0min⁡_ = 12.2 mK 2LS tunneling parameter. We have fitted all data with a more realistic Δ_0min⁡_ = 3.9 mK and best fit parameters from Table 1 using (8) and (14) (driving frequency *ω* = 1 kHz).

Figure 7 (inset) shows the behavior of the *T*-dependent part of *ϵ*′(*T*, *ω*), Δ*ϵ*′/*ϵ*′ = [*ϵ*′(*T*) − *ϵ*′(*T*_0_)]/*ϵ*′(*T*_0_), (where *T*_0_(*ω*) is a characteristic minimum) for pure vitreous SiO_2_ (Spectrosil). It can be seen that linear regimes in −ln⁡*T* for *T* < *T*_0_ and +ln⁡*T* for *T* > *T*_0_ are observed, and roughly with slopes *S*_−_ = −2*S* and *S*_+_ = +*S* > 0, or in a −2 : 1 ratio. According to the 2LS-STM, in fact, we have the expressions [14–18, 51]

(8) Δϵ′ϵ′|2LS=Δϵ′ϵ′|2RES+Δϵ′ϵ′|2REL,Δϵ′ϵ′|2RES=2P−p02¯3ϵ0ϵr∫zmin⁡zmax⁡dzz1−(Δ0min⁡2kBTz)2tanhz,Δϵ′ϵ′|2REL=P−p02¯3ϵ0ϵr×∫zmin⁡zmax⁡dz∫τmin⁡τmax⁡dττ1−τmin⁡τ×cosh⁡−2(z)11+ω2τ2,

where we neglect (for low *ω*) the frequency dependence in the RES part, where *z*_min⁡,max⁡_ = Δ_0min⁡,max⁡_/2*k*_*B*_ *T* and where *τ* is the phenomenological 2LS relaxation time given by (with *E* = 2*k*_*B*_ *Tz*) [17, 18]:

(9) $$\begin{array}{c} \tau^{-1}=\frac{E\Delta_{0}^{2}}{\gamma }\tanh\left(\frac{E}{2k_{B}T}\right). \end{array}$$

In these expressions, Δ_0min⁡_ and Δ_0max⁡_ are Δ_0_'s phenomenological bounds, *γ* is an elastic material parameter of the solid, and *τ*_min⁡_^−1^ = *E* ^3^/*γ*tanh(*E*/2*k*_*B*_ *T*), *τ*_max⁡_^−1^ = *E*Δ_0min⁡_^2^/*γ*tanh(*E*/2*k*_*B*_ *T*). $\overset{-}{P}$ (containing the 2LS volume concentration, *x*_2LS_) is the probability per unit volume and energy that a 2LS occurs in the solid (it appears in (2)) and $\bar{p_{0}^{2}}$ is the average square 2LS electric dipole moment. Moreover, the strategy of dielectric relaxation theory has been adopted, whereby the full complex dielectric constant *ϵ*(*T*, *ω*) has been written as, for *ωτ* ≪ 1 [51, 52],

(10) $$\begin{array}{c} \epsilon \left(T,\omega \right)=\epsilon_{\text{RES}}^{\prime }\left(T\right)+\epsilon_{\text{REL}}^{\prime }\left(T\right)\frac{1}{1+i\omega \tau }; \end{array}$$

the subscripts RES and REL refer to the zero relaxation-time resonant and relaxational contributions to the linear response *ϵ*′ at zero frequency, respectively.

Presently, from expressions (8) we deduce that: (1) the so-called resonant (RES) contribution has the leading behavior

(11) $$\begin{array}{c} {\frac{\Delta \epsilon^{\prime }}{\epsilon^{\prime }}|}_{2\text{RES}}≃\{\begin{array}{cc} -\frac{2}{3}\frac{\overset{-}{P}\bar{p_{0}^{2}}}{\epsilon_{0}\epsilon_{r}}\ln\left(\frac{2k_{B}T}{\Delta_{0\max}}\right) & \text{if}\;\;T<\frac{\Delta_{0\max}}{2k_{B}},\\ 0 & \text{if}\;\;T>\frac{\Delta_{0\max}}{2k_{B}}; \end{array} \end{array}$$

(2) the relaxational (REL) contribution has, instead, the leading behavior

(12) $$\begin{array}{c} {\frac{\Delta \epsilon^{\prime }}{\epsilon^{\prime }}|}_{2\text{REL}}≃\{\begin{array}{cc} 0 & \text{if}\;\;\omega \tau_{\min}\gg 1,\\ \frac{1}{3}\frac{\overset{-}{P}\bar{p_{0}^{2}}}{\epsilon_{0}\epsilon_{r}}\ln\left(\frac{2k_{B}T}{\Delta_{0\min}}\right) & \text{if}\;\;\omega \tau_{\min}\ll 1. \end{array} \end{array}$$

Thus, the sum of the two contributions has a V-shaped form, in a semilogarithmic plot, with the minimum occurring at a *T*_0_ roughly given by the condition *ωτ*_min⁡_(*k*_*B*_ *T*)≃1, or *k*_*B*_ *T*_0_(*ω*)≃((1/2)*γω*)^1/3^. *ϵ*_0_ *ϵ*_*r*_ is here the bulk of the solid's dielectric constant, and we see that a −2 : 1 characteristic behavior is justified by the STM with the *T* > *T*_0_ slope given by $S=\overset{-}{P}\bar{p_{0}^{2}}/3\epsilon_{0}\epsilon_{r}$.

**Figure 8:**
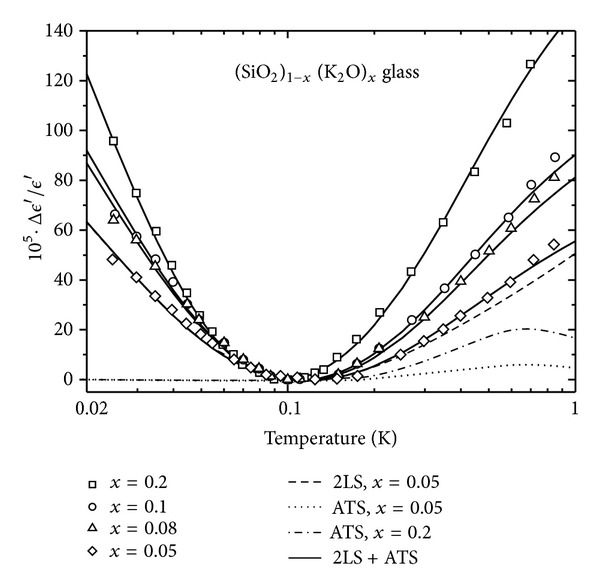
Dielectric signature of mixed (SiO_2_)_1−*x*_(K_2_O)_*x*_ glasses as function of *T* and *x* [24]. Fitting parameters from Table 1 using (8) and (14) from our theory (driving frequency *ω* = 10 kHz).

This behavior is observed in pure *a*-SiO_2_ [53] (Figure 7 (inset), with the fitting parameters of Table 1, *x* = 0, from our own best fit to (8)). However, in most multicomponent glasses one more often observes a V-shaped curve with a (roughly) −1 : 1 slope ratio. Figure 7 (main) shows this phenomenon for the multisilicate AlBaSiO glass (in fact, a MAS-type ceramic-glass), which has been extensively investigated in recent times due to its unexpected magnetic field response [6, 7, 9–12, 21]. Also, Figure 8 shows the remarkable behavior of the dielectric constant versus *T* for the glasses of composition (SiO_2_)_1−*x*_(K_2_O)_*x*_ containing a molar concentration *x* of alkali oxide [24]. It is seen that a *S*_−_/*S*_+_ slope ratio of roughly −1 : 1 is observed, with the slope definitely changing with *x* (and faster for *T* > *T*_0_). These data from the Anderson group [24], thus far unexplained by the 2LS-STM, call for an extension of the accepted STM, and we show below that a simple explanation can be given in terms of the very same ATS that have been justified in Section 2 and advocated by one of us in order to explain the magnetic response of AlBaSiO and other multicomponent glasses [21]. In view of the interest for these materials in low-*T* metrology, and on fundamental grounds, such explanation appears overdue to us. Moreover, “additional” TS (beside the standard 2LS) of the type here advocated were already called for in [24] and other theoretical papers [55–57].

For the multiwelled (3LS, in practice) Hamiltonian (3), we have *n*_*w*_ = 3 low-lying energy levels, with *ℰ*_0_ < *ℰ*_1_ ≪ *ℰ*_2_. In the *E*_*i*_ → 0 and $D\equiv \sqrt{E_{1}^{2}+E_{2}^{2}+E_{3}^{2}}\ll D_{0}$ limits (due to the chosen near-degenerate distribution, (6)), we can approximate the *n*_*w*_ = 3-eigenstate system through an *effective 2LS* (though sensitive to all three well asymmetries and their distribution) having gap Δ*ℰ* = *ℰ*_1_ − *ℰ*_0_:

(13) $$\begin{array}{c} \lim\Delta ℰ≃\sqrt{E_{1}^{2}+E_{2}^{2}+E_{3}^{2}}\equiv D. \end{array}$$

We have also exploited the condition *E*_1_ + *E*_2_ + *E*_3_ = 0. Using the theory of [21] to work out the 3LS contributions to *ϵ*_RES_′ and *ϵ*_REL_′, we arrive at the following expressions for the contribution to the dielectric anomaly from the advocated ATS:

(14) Δϵ′ϵ′|ATS=Δϵ′ϵ′|ARES+Δϵ′ϵ′|AREL,Δϵ′ϵ′|ARES=πP~∗p12¯3ϵ0ϵrDmin⁡∫1∞dyy2tanh(Dmin⁡2kBTy),Δϵ′ϵ′|AREL=πP~∗p12¯2ϵ0ϵrDmin⁡(Dmin⁡2kBT)×∫1∞dyycosh⁡−2(Dmin⁡2kBTy)11+ω2τAmax⁡2.

Here, we have again neglected, for low-*ω*, the frequency dependence in the RES part; we have put *y* = *D*/*D*_min⁡_, and *τ*_*A*max⁡_ is the largest phenomenological ATS relaxation time given by [59]

(15) $$\begin{array}{c} \tau_{A\max}^{-1}=\frac{D^{5}}{\Gamma }\tanh\left(\frac{D}{2k_{B}T}\right). \end{array}$$

Moreover, *D*_min⁡_ is the lowest energy gap of the multilevel ATS, Γ is another appropriate elastic constant, and ${\tilde{P}}^{*}$ is the (slightly renormalised) probability per unit volume (after inclusion of *x*_ATS_) that an ATS occurs within the NM-pockets and channels, with $\bar{p_{1}^{2}}$ the average square ATS dipole moment. ${\tilde{P}}^{*}$ and P* are so related:

(16) $$\begin{array}{c} {\tilde{P}}^{*}=P^{*}\ln\left(\frac{D_{0\max}}{D_{0\min}}\right). \end{array}$$

*D*_0min⁡_ and *D*_0max⁡_ being *D*_0_'s lower and upper bounds, respectively. This description is intimately linked to the chosen distribution function, (6), for these ATS which is favoring near-degenerate energy gaps *D* bound from below by *D*_min⁡_. In turn, this produces an overall density of states given by ([21], for *B* = 0):

(17) $$\begin{array}{c} g\left(E\right)=g_{2\text{LS}}+g_{\text{ATS}}\left(E\right)≃2\overset{-}{P}+\frac{2\pi {\tilde{P}}^{*}}{E}\theta \left(E-D_{\min}\right), \end{array}$$

and that is now roughly of the form advocated by Yu and Legget [25–27] and by some other preceeding authors (e.g., [60]) to explain anomalies not accounted for by the standard 2LS-TM. *θ*(*x*) is the step function.

Manipulation of the expressions in (14) shows that (1) the RES contribution from the ATS has the leading behavior (note that for *T* < *D*_min⁡_/2*k*_*B*_,  *ϵ*′|_ARES_ is roughly a constant)

(18) $$\begin{array}{c} {\frac{\Delta \epsilon^{\prime }}{\epsilon^{\prime }}|}_{\text{ARES}}≃\{\begin{array}{cc} 0 & \text{if}\;\;T<\frac{D_{\min}}{2k_{B}},\\ \frac{\pi {\tilde{P}}^{*}\bar{p_{1}^{2}}}{6\epsilon_{0}\epsilon_{r}k_{B}T}\ln\left(\frac{2k_{B}T}{D_{\min}}\right) & \text{if}\;\;T>\frac{D_{\min}}{2k_{B}}; \end{array}\; \end{array}$$

(2) the REL contribution is, instead, characterised by the leading form

(19) $$\begin{array}{c} {\frac{\Delta \epsilon^{\prime }}{\epsilon^{\prime }}|}_{A\text{REL}}≃\{\begin{array}{cc} 0 & \text{if}\;\;\omega \tau_{A\max}\gg 1,\\ \frac{\pi {\tilde{P}}^{*}\bar{p_{1}^{2}}}{\epsilon_{0}\epsilon_{r}k_{B}T}\ln\left(\frac{k_{B}T}{D_{\min}}\right) & \text{if}\;\;\omega \tau_{A\max}\ll 1. \end{array} \end{array}$$

Thus, the V-shaped semilogarithmic curve is somewhat lost. However, adding the 2LS (8) and ATS (14) contributions together, one does recover a rounded V-shaped semilog with a slope *S*_−_≃−2*S* basically unchanged for *T* < *T*_0_ and an augmented slope *S*_+_ = *S* + *S*_ATS_ for *T* > *T*_0_ with $S_{\text{ATS}}=7\pi {\tilde{P}}^{*}\bar{p_{1}^{2}}/6\epsilon_{0}\epsilon_{r}k_{B}T$  that for  *T* < *D*_min⁡_/*k*_*B*_ may approach 2*S* and thus (qualitatively) explain a −1 : 1 slope ratio.

**Figure 9:**
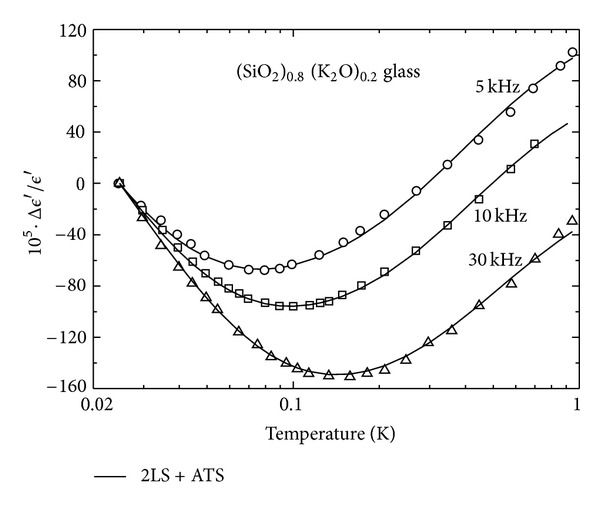
Dielectric signature of mixed (SiO_2_)_1−*x*_(K_2_O)_*x*_ glasses as function of *T* and *ω* for *x* = 0.2 [24]. Fitting parameters from Table 1 using (8) and (14) from our theory.

**Figure 10:**
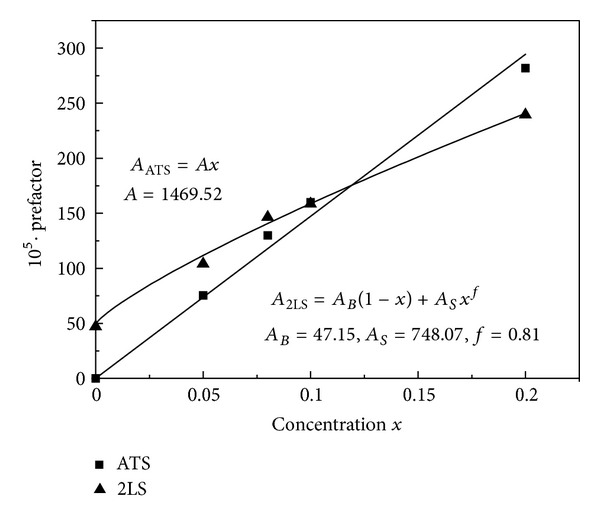
The 2LS and ATS dimensionless prefactor parameters (×10^5^) for all glasses (from Table 1) as a function of *x*. Our data fit well with our theoretical expectations (full lines).

**Figure 11:**
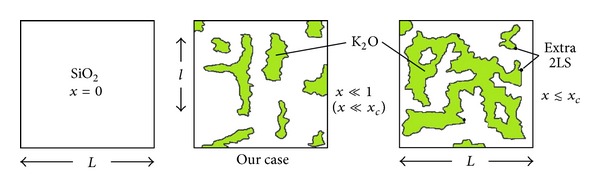
(Color online) A cartoon of the fractal (presumably percolating) geometry of the NM-pockets and channels (green); these NM-clusters grow with increasing *x*.

We have fitted the full expressions (8) and (14) to the data for AlBaSiO in Figure 7 (main) and to the *x*-dependent data for (SiO_2_)_1−*x*_(K_2_O)_*x*_ in Figures 8 and 9, obtaining in all cases very good agreement between theory and experiments [24].  Figure 9 shows the fit of our theory to the frequency-dependent data for *x* = 0.2. In all of these best fits, we have kept the value of Δ_0min⁡_ = 3.9 mK fixed, as obtained from our pure SiO_2_ fit, and the value of *D*_min⁡_ also independent of *x* and *ω*. The idea is that these parameters are rather local ones and should not be influenced by NF/NM dilution. Table 1 gathers the values of all the (2LS and ATS) parameters used for our best fits and Figure 10 shows the dependence of the prefactors (containing *x*_2LS_ in $\overset{-}{P}$ and *x*_ATS_ in ${\tilde{P}}^{*}$) with *x*. It can be seen that, as expected, the ATS prefactor $A_{\text{ATS}}=\pi {\tilde{P}}^{*}\bar{p_{1}^{2}}/\epsilon_{0}\epsilon_{r}D_{\min}$ scales linearly with *x*, an excellent confirmation that the “additional”  TS of [24, 55–57] are those ATS, proposed by us and modelled as 3LS, forming near and inside the microcrystallites that may nucleate within the NM-pockets and channels. It can be seen, instead, that the 2LS prefactor $A_{2\text{LS}}=\overset{-}{P}\bar{p_{0}^{2}}/\epsilon_{0}\epsilon_{r}$ of our fits also increases, though less rapidly, with increasing *x* (a decrease like 1 − *x* would be expected). We propose (adopting a NF-, NM-cluster percolation picture) that new, “dilution-induced” 2LS form with alkali mixing near the NF/NM interfaces of the NF percolating cluster(s) as *x* is increased from 0. This reasoning leads to the expression *A*_2LS_ = *A*_bulk_(1 − *x*) + *A*_surf_ *x* ^*f*^ for the 2LS prefactor, with *A*_bulk_,  *A*_surf_,  and *f* fitting parameters. Our best fit leads to the value *f* = 0.81, in fair agreement with the euristic expression

(20) $$\begin{array}{c} f=1-\left(D-D_{s}\right)\nu , \end{array}$$

(where *D* is the fractal dimension of the percolating cluster, *D*_*s*_ with *D*_*s*_ ≤ *D* is that of its “bridging” surface (not necessarily the hull) and *ν* is the connectedness length's exponent) that one would deduce from elementary fractal or percolation theory (see, e.g., [61, 62]). *D*_*s*_ is the fractal dimension of that part of the NM random-cluster's surface where formation of TS takes place and we expect 2 ≤ *D*_*s*_ ≤ *D*. It is indeed reasonable to expect new TS to be forming at these NM/NF random interfaces, for these are surfaces of chemical discontinuity in the material. The above expression is derived as follows. Imagine (as is shown in the cartoons in Figure 11) the NM-clusters percolating through the NF-bulk with a site concentration *x*, so that their volume scales like *𝒱* ~ *ℓ* ^*D*^, where *ℓ* ~ *x* ^*ν*^ is their typical linear size. The number of 2LS on the surface of these clusters will scale like *N*_2LS_^(*s*)^ ~ *xℓ* ^*D*_*s*_^ and so their density like *N*_2LS_^(*s*)^/*𝒱* ~ *xx* ^(*D*_*s*_−*D*_*f*_)*ν*^ = *x* ^*f*^ with the given expression, (20), for *f*. If we consider clusters of 2D percolation and assume *D*_*s*_ = *D*_*h*_ = 7/4 (the fractal dimension of the hull of the spanning cluster), then with *D* = 91/48 and *ν* = 4/3 [61, 62] we would get *f* = 29/36 = 0.8055. More realistically, on the assumption of percolating 3D NM-clusters in the mixed glasses, we can make use of the values [61–63] *D*≃2.52, *D*_*s*_ = *D*_*h*_ = 2.14, and *ν*≃0.88 to arrive at the value *f* = 0.67 using (20) (We are well aware that the quoted fractal dimensions apply to percolation clusters, strictly, only at the percolation threshold *x* = *x*_*c*_. Our attitude is that fractal-type clusters can be used to model the NM-lumps from the good crystal-formers even for *x* < *x*_*c*_ and with non-integer fractal dimensions. This assumption failing, we have no explanation for the extracted non-zero value of the *f*-exponent and *A*_surf_-prefactor.). It is however not at all clear where, at the NM/NF fractal interfaces, the new 2LS will form (i.e., what the exact definition of *D*_*s*_ ought to be: hull surface sites, screening sites, dead-end sites, etc.). If all of the hull sites are involved, then for 3D *x* = *x*_*c*_ percolation *D*_*s*_ = *D* and one then expects *f* = 1. Thus, this new phenomenology opens a tantalizing new investigation avenue for research on the applications of fractal theory to low-*T* physics. At the same time, the knowledge of which type of NM/NF fractal interface sites are involved in the TS-formation would greatly improve our understanding about the microscopic nature of the TS (see also [28]).

## 4. Predictions for the Heat Capacity

We now come to the explanation of the, also rather anomalous, heat-capacity data for the mixed glasses (SiO_2_)_1−*x*_(K_2_O), reported in [24] as a function of  *T* and for different *x*. The heat capacity's low-temperature dependence in zero magnetic field is, for pure glasses, usually given by the following expression:

(21) $$\begin{array}{c} C_{p}\left(T\right)=B_{\text{ph}}T^{3}\;+B_{2\text{LS}}T. \end{array}$$

**Figure 12:**
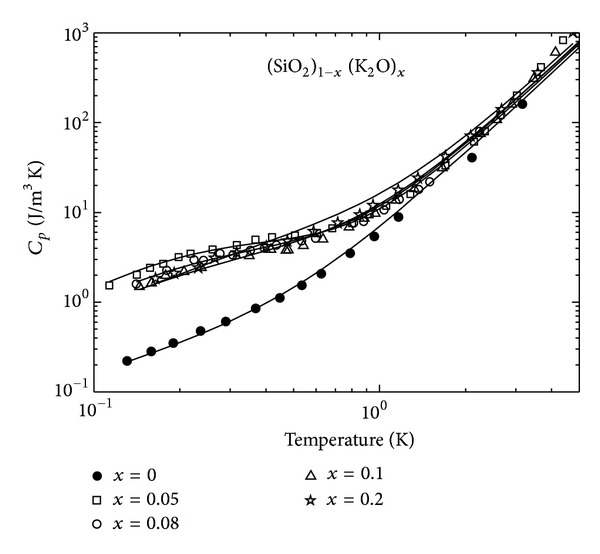
The temperature dependence of the heat capacity for *a*-SiO_2_ (black circles) and for the (SiO_2_)_1−*x*_(K_2_O)_*x*_ glasses [24]. The full lines are our theoretical curves, as generated by (31).

The first term accounts for the Debye-type contribution from the acoustic phonons and dominates above 1 K; the second term is usually attributed to the low-energy excitations specific of all vitreous solids—the tunneling 2LS. *B*_ph_ and *B*_2LS_ are material-dependent constants. This expression describes well the experimental data for pure silica glass at zero field (Figure 12, black circles: *x* = 0 with fit parameters from Table 2), but it fails for the multicomponent glasses, like AlBaSiO, BK7, Duran (see, e.g., [21] and references therein) and for the mixed glasses (SiO_2_)_1−*x*_(K_2_O)_*x*_ for *x* > 0 [24].

Typically, the heat capacity's experimental data for the multicomponent glasses in zero field denote a kind of “shoulder” at intermediate-low temperatures. This suggests a density of states, for at least some of the independent TS in the glass, of the form *g*(*E*) ∝ 1/*E*, in contrast to the standard 2LS-TM prediction, *g*(*E*)≃ const., which ensues from the standard TM distribution of parameters. Indeed, this 1/*E* contribution to the DOS was the very first observation that has led to the hypothesis of the ATS formulated in [21].

To find out the precise expression for the heat capacity due to the ATS, we make use of the 3LS formulation for the ATS described in [21] and in more detail in Section 2. The heat capacity is determined from the second derivative of the free energy with respect to temperature:

(22) $$\begin{array}{c} C_{p}^{\text{ATS}}\left(T\right)=-T\frac{\partial^{2}F_{\text{ATS}}\left(T\right)}{\partial T^{2}}, \end{array}$$

where *F*_ATS_(*T*) is the free energy of the ATS given by, if we neglect the third, highest energy level in the spectrum of Hamiltonian (3) (effective 2LS approximation):

(23) FATS(T)=−kBTln⁡(e−ℰ0/kBT+e−ℰ1/kBT)=−kBTln⁡(2cosh⁡(E2kBT)),

with *E* = *ℰ*_1_ − *ℰ*_0_. The heat capacity is then obtained by averaging over the parameter distribution, or, equivalently, by a convolution with the DOS:

(24) $$\begin{array}{c} C_{p}^{\text{ATS}}\left(T\right)=k_{B}\int_{0}^{\infty }dEg_{\text{ATS}}\left(E\right){\left(\frac{E}{2k_{B}T}\right)}^{2}\cosh^{-2}\left(\frac{E}{2k_{B}T}\right), \end{array}$$

where density of states *g*_ATS_(*E*) has the following form [21]:

(25) gATS(E)=∫dD∫dD0P(D,D0)δ(E−D)≃{2P∗Eif  E>Dmin⁡,0if  E<Dmin⁡

and *D*_min⁡_ is the lower cutoff. The final expression for the ATS heat capacity results in [21]:

(26) CpATS(T)=BATS[ln⁡(2cosh⁡(Dmin⁡2kBT))   −Dmin⁡2kBTtanh(Dmin⁡2kBT)],

where the prefactor for the ATS is $B_{\text{ATS}}=2\pi {\tilde{P}}^{*}k_{B}n_{\text{ATS}}\rho (x)$, ${\tilde{P}}^{*}$ as in Section 3, *n*_ATS_ being the ATS mass concentration, and *ρ*(*x*) the glass' mass density. Of course, *x*_ATS_ = *n*_ATS_ *ρ*(*x*). For *k*_*B*_ *T* ≳ *D*_min⁡_, this is indeed roughly a constant and gives the observed “shoulder” in *C*_*p*_(*T*) when the contribution *B*_ph_ *T* ^3^ (from virtual phonons) as well as the STM linear term *B*_2LS_ *T* are taken into account.

Both prefactors, for the 2LS and ATS contributions, are dependent on the molar concentration *x* of alkali-oxide, just as we found in Section 3 for the prefactors of the dielectric constant: *B*_2LS_≃*B*_bulk_(1 − *x*) + *B*_surf_ *x* ^*f*^, *B*_ATS_≃*Bx*. Also *B*_ph_ requires to be reevaluated. With increasing K_2_O molar concentration *x* for the (SiO_2_)_1−*x*_(K_2_O)_*x*_ glass, the number of phonons from the NM-component (K_2_O in this case) increases linearly with the concentration *x*, and for the NF-component (SiO_2_) it should also decrease linearly, like (1 − *x*). Just as we assumed in the previous section, there are fractal/percolation effects between the NM- and NF-clusters, which makes room for some percolation clusters' interfaces where the phonons also might contribute somehow with a term proportional to *C*_ph_ *x* ^*f*^ (*C*_ph_ being an *x*-independent constant).

For these glasses, moreover, a nonnegligible concentration of Fe^3+^ (or, according to coloring, Fe^2+^) impurities is reported, a side effect of the industrial production process. Estimates give 102 ppm for AlBaSiO and 126 ppm for Duran, 6 ppm for BK7, 100 ppm for Pyrex 7740, and 12 ppm for Pyrex 9700 (see, e.g., the discussion in [21]). All glasses may, indeed, contain some [FeO_4_]^0^ impurity-substitution F-centers (in the glass, similar to a liquid, in concentrations however much, much lower than the nominal Fe bulk concentrations [43]). The Fe^3+^ cation and the O^2−^ anion, on which the hole is localized (forming the O^−^ species, i.e., the O^2−^ + hole subsystem), form a bound small polaron. In this configuration, the Fe^3+^ cation is subject to a crystal field with an approximate *C*_3_ symmetry axis along the Fe^3+^-O  ^−^ direction. This axis plays a quantization role for the Fe^3+^ electronic spin. The hole is assumed to be tunneling between two neighboring oxygen ions, switching the quantization axis between two directions, and therefore entangling its spin states. This is likely to give some tiny contribution to the heat capacity, and we should, therefore, also take it here into account [64]. The spin Hamiltonian of the [FeO_4_]^0^ F-center is *H*_*s*−*S*_ = *V*_*z*_ *s*_*z*_ *S*_*z*_, where *V*_*z*_ is the principal value of the dipole interaction matrix and *s*_*z*_ and *S*_*z*_ are the spin operators of the hole and of the Fe^3+^ ion, respectively. In the absence of a magnetic field, there are only two low-lying energy levels: *E*_1,2_ = ±(5/4)|*V*_*z*_|. The unknown distribution function G(*V*_*z*_) must approach zero when its argument approaches either zero or infinity and have a maximum at a definite argument value *V*_0_. The simplest one-parameter function displaying such properties is a Poisson distribution:

(27) $$\begin{array}{c} G\left(V_{z}\right)=\frac{4V_{z}^{2}}{V_{0}^{3}}\exp\left(-\frac{2V_{z}}{V_{0}}\right),\;V_{z}\in \left(-\infty ;0\right],\;\;V_{0}<0. \end{array}$$

The contribution from the [FeO_4_]^0^ ensemble to the heat capacity is, as usual,

(28) $$\begin{array}{c} C_{\text{F}{\text{e}}^{3+}}\left(T\right)=-T\frac{\partial^{2}F_{\text{F}{\text{e}}^{3+}}}{\partial T^{2}}, \end{array}$$

where *F*_Fe^3+^_(*T*) is the free energy of the [FeO_4_]^0^ ensemble, that one evaluates as

(29) FFe3+=−kBTln⁡(e−E1/kBT+e−E2/kBT)=−kBTln⁡(2cosh⁡(E2kBT));

here *E* = (5/4)|*V*_*z*_|. Using said distribution function for *G*(*V*_*z*_), (27), as well as the expression for *C*_Fe^3+^_(*T*) from *F*_Fe^3+^_(*T*), one can obtain an expression for the heat capacity from the trace [FeO_4_]^0^ centres in the glass, and which should be added to the total heat capacity *C*_*p*_:

(30) CpFe3+(T)=ρ(x)njkB ×∫0∞dVz(E2kBT)2cosh⁡−2(E2kBT)G(Vz)=ρ(x)njkB∫0∞dVz25Vz416T21V03e(−2Vz/V0) ×cosh⁡−2(5Vz8kBT),

where *n*_*j*_ = *x*_*j*_/*ρ*(*x*) is the mass concentration of the tiny amount of Fe^3+^ ions (a very small fraction of the total bulk Fe-concentration) substituting the Si^4+^ in the network.

Hence, the total heat capacity will be the sum of all these contributions: (21), (26), and (30) (Regarding the role of the Fe-impurities at zero magnetic field, this was totally overlooked in [21] where, to provide a good fit of the data, the existence of a weak, stray magnetic field was wrongly advocated.):

(31) $$\begin{array}{c} C_{p}\left(T\right)=B_{\text{ph}}T^{3}+B_{2\text{LS}}T+C_{p}^{\text{ATS}}\left(T\right)+C_{p}^{\text{F}{\text{e}}^{3+}}\left(T\right). \end{array}$$

Making use of expression (31), we have fitted the experimental data for the heat capacity of the (SiO_2_)_1−*x*_(K_2_O)_*x*_  glasses from [24]. In order to fit the pure *a*-SiO_2_ data, we use only formula (21), that fits the pure silica's data well within the 2LS-STM.

The heat capacity *C*_*p*_(*T*, *x*) data [24] for the (SiO_2_)_1−*x*_(K_2_O)_*x*_ glasses were obtained using a signal-averaging technique and for these samples the data are presented in Figure 12. As one can see, the heat capacity for the (SiO_2_)_1−*x*_(K_2_O)_*x*_ glasses at low temperatures is larger than that for pure silica glass, as is typical for the multicomponent glasses, already with the smallest 5% concentration of K_2_O. The heat capacity decreases and then again increases with increasing molar concentration *x* of K_2_O. The additional heat capacity arises from the addition of ATS in the K_2_O NM-clusters and also from the presence of Fe^3+^ impurities, contained in small (and unknown) concentrations, but contributing to the low- and middle-range of the temperature dependence.

**Figure 13:**
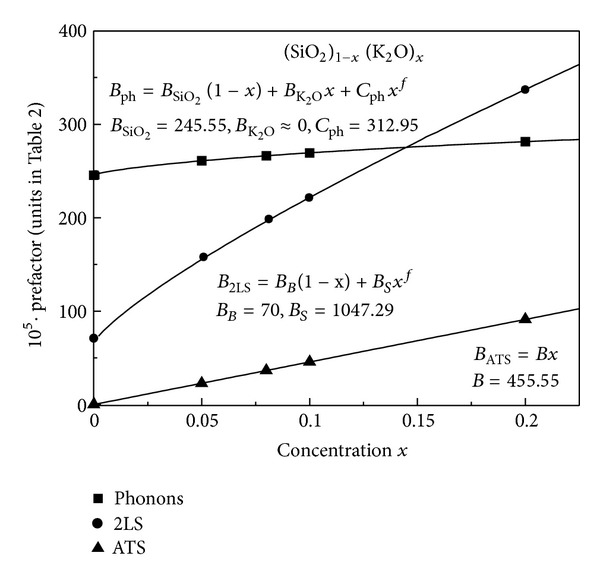
The 2LS and ATS prefactor parameters (×10^8^) for all glasses (from Table 2) as a function of *x*. The experimental data fit well with our theoretical expectations with *f* = 0.81 (full lines).

Both prefactors, for 2LS and ATS, are indeed dependent on the molar concentration *x* from our data analysis, and in the same way as we did in Section 3 we have fitted the extracted prefactors with the forms: *B*_2LS_≃*B*_bulk_(1 − *x*) + *B*_surf_ *x* ^*f*^, *B*_ATS_≃*Bx* (*B* being some constant). These dependencies are shown in Figure 13. Also *B*_ph_ is found to change by increasing the concentration *x* of the good crystal-former, K_2_O, and in the way we anticipated.

With increasing concentration *x*, for the (SiO_2_)_1−*x*_(K_2_O)_*x*_ glass, the number of phonons from the NM-component (K_2_O) increases linearly with the concentration, and for the NF-component (SiO_2_), it should be decreasing linearly like (1 − *x*). As we reasoned for the dielectric constant, there are percolation mixing effects between the NM- and the NF-systems, which create percolation clusters and their NF/NM interfaces where phonons also might be populated in a way proportional to *C*_ph_ *x* ^*f*^. As it turns out, the very same value *f* = 0.81 can be extracted from all our fits, just as was done in Section 3 for the dielectric constant data.

## 5. Summary and Conclusions

We have demonstrated that there is direct evidence in zero magnetic field already for the existence of multiwelled ATS (modelled as tunneling 3LS) and with the new distribution function advocated to explain the magnetic field effects in the multicomponent glasses (see [21]). The relevance of near-degenerate multiwelled TS in glasses is a new and unexpected finding in this field of research. Our work predicts, in particular, that the magnetic response of the mixed alkali-silicate glasses should be important and scale like the molar alkali concentration *x*. At the same time, the −1 : 1 slope-ratio problem of the standard TM in comparison with experimental data for *ϵ*′(*T*) has been given a simple explanation in terms of our two-species tunneling model. The main result of this work is that the concentration *x*_ATS_ (absorbed in ${\tilde{P}}^{*}$ and thus in the *A*_ATS_- and *B*_ATS_-prefactors) of ATS indeed scales linearly with *x* for both *ϵ*′(*T*, *x*) and *C*_*p*_(*T*, *x*). This is supported by our analysis of the existing experimental data, [24], very well indeed. Our analysis is, in our view, strong evidence that the ATS are nesting in the NM-clusters of the good crystal-formers. Our fractal modeling of the phase-separation NF/NM cluster interfaces in the multicomponent glasses gives a strong indication that the TS are forming in correspondence to the chemical discontinuities in the structure of amorphous materials. The justification of our mathematical modeling implies the existence of incipient crystallites in all amorphous solids, where the relevant degrees of freedom appear to be correlated over decades or even hundreds of atomic spacings. This cooperativity now seems to be a commonplace occurrence in the glassy state at all temperatures below *T*_*g*_.

Using the results of this analysis (and for AlBaSiO the results of the experimental data analysis in a magnetic field [21]), we can estimate the value of the dipole moment associated with the ATS, $p_{\text{eff}}=\sqrt{\bar{p_{1}^{2}}}$. For AlBaSiO, using the value of ${\tilde{P}}^{*}$ extracted from *C*_*p*_ [21] and that of *A*_ATS_ given in Table 1, we extract *p*_eff_ = 0.41 D. For (SiO_2_)_1−*x*_(K_2_O)_*x*_, we notice from the definitions in Section 3 that the ratio of the dielectric and heat capacity prefactors

(32) $$\begin{array}{c} \frac{A_{\text{ATS}}}{B_{\text{ATS}}}=\frac{\rho \left(x\right)}{2\epsilon_{0}\epsilon_{r}k_{B}D_{\min}{{\overset{-}{p}}_{1}}^{2}}, \end{array}$$

is almost independent of the K_2_O concentration *x*. From our extracted values in Tables 1 and 2 and the measured values of *ρ*(*x*) [24], we estimate *p*_eff_ = 0.045 D for the mixed glasses, independently of *x*! Considering the elementary atomic electric-dipole's value is *ea*_0_ = 2.54 D, these small values of *p*_eff_ for the ATS confirm that their physics must come from the coherent (or correlated) tunneling of small ionic clusters (the very same origin for the large values of *D*_min⁡_ and for *D*_0min⁡,max⁡_; see the appendix). Indeed, a cluster of *N* coherently tunneling particles has a dipole moment *p*_eff_ = |∑_*i*=1_^*N*^ **p**_*i*_| that can become much smaller than *ea*_0_ (the order of magnitude of each |**p**_*i*_| in the sum) as *N* grows large. The fact, that we extract values of *p*_eff_ much smaller than *ea*_0_, confirms the picture of a correlated tunneling cluster in the *B* = 0 case already.

It is noteworthy that several papers from the Anderson group have proved that the addition of any NM-species in a networking pure glass causes significant (and thus far unexplained) deviations from the predictions of the 2LS-STM [24, 65, 66]. We have explained the origin of these deviations for *C*_*p*_(*T*, *x*) as well as for *ϵ*(*T*, *x*). However, experiments do show that the thermal conductivity *κ*(*T*, *x*) ∝ *T* ^2^ remains (below 1 K) remarkably universal and composition independent [24]. This is connected with the superuniversality of the internal friction coefficient, *Q* ^−1^, in the cold glasses; these and other remarkable findings will be addressed elsewhere within the context of our approach.

In summary, we have shown that there is direct evidence in zero magnetic field already for the multiwelled ATS advocated to explain the magnetic field effects in the multicomponent glasses. Similar *x*-dependent phenomena are to be expected for the low-*T* anomalies of the MAS-type ceramic-glass of composition (SiO_2_)_1−*x*_(MgO)_*x*_, which should also respond to the magnetic field (Experiments on these glasses, using different isotopes Mg^24^-Mg^26^ and Mg^25^, could also serve to confirm the nuclear quadrupole explanation for the magnetic effects.) (just like the mixed alkali-silicates of this work should). One may remark, at this point, that any extension of the 2LS-STM enlarging the adjustable-parameter space is bound to improve agreement with the experimental data. In this paper, we have shown that it was not just a matter of quantitative agreement, but qualitative as well. Whilst agreeing that the TM remains unsatisfactory, we stress that it is the only approach we know of, which is versatile enough to allow for an interesting explanation of rather puzzling phenomena at low-*T* in the real glasses. Furthermore, our two-species, multilevel TS model has been able to consistently explain a good number of different experimental data [21, 59]. It cannot be a mere coincidence that the same phenomenological model, with rather similar material parameters in different experiments, is capable of explaining so much new physics. Far from being an ad hoc model, our approach reveals the intimate microscopic structure of the real glasses, which cannot be considered as being homogeneously disordered anymore, and this must have some important consequences also for a better understanding of the mechanisms underlying the glass transition.

As for the possibility of estimating the size and density of the incipient crystals in glasses from our theory, we remark that the simplified geometric-averaging procedure adopted for the physics of the ATS so far [21] does not allow anything more than an estimate of the P* parameter (~1.97 × 10^17^ cm^−3^ for AlBaSiO [21], this being in fact the value of *x*_ATS_ P*, P* being the unknown dimensionless parameter of the ATS distribution in (6)). However, the geometric-averaging procedure should be performed in two stages (within the incipient microcrystals first and then within the glassy matrix in which the crystallites are embedded) at the price of making the theory considerably more complicated. When this is done, with a more efficient and complete theoretical formulation, then information on the size distribution of the incipient crystallites could be gained from further low-*T* experiments in magnetic fields and at different controlled compositions.

## Figures and Tables

**Figure 14:**
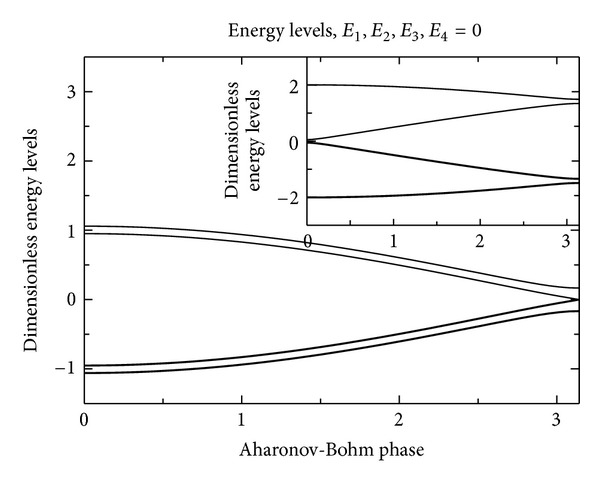
(Main) Variation with the magnetic Aharonov-Bohm phase *ϕ* of the energy spectrum (units *D*_2_ = 1) for the case *D*_1_ = 0 and a choice of *E*_1_, *E*_2_, *E*_3_, *E*_4_ with $\sqrt{E_{1}^{2}+E_{2}^{2}+E_{3}^{2}+E_{4}^{2}}/D_{2}=0.01$. This is to be compared with the 3LS energy spectrum, Figure 5. (Inset) The energy spectrum in the opposite case, *D*_2_ = 0, and a choice of *E*_1_, *E*_2_, *E*_3_, *E*_4_ with *D*_1_ about 100 times stronger.

**Figure 15:**
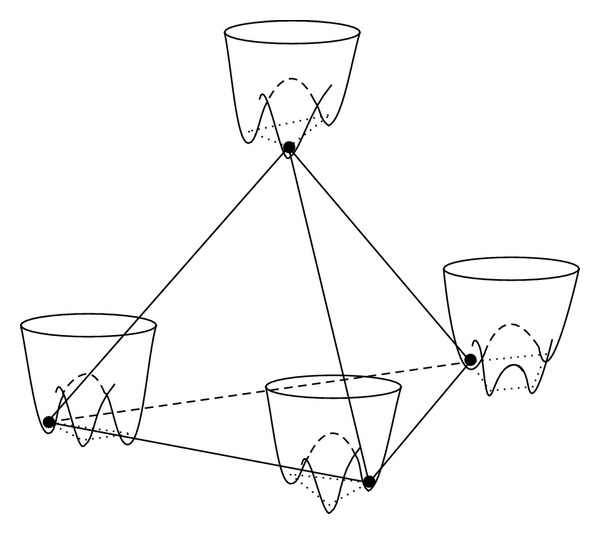
A cluster of *N* = 4 weakly interacting (real) tunneling particles that is being replaced with a (fictitious) single 3LS (Figure 4(c)) having renormalised parameters according to ((A.9)) and ((A.10)).

**Table 1:** Extracted parameters for the glasses; K-Si stands for the (SiO_2_)_1−*x*_(K_2_O)_*x*_ glasses. In all of the best fits, we have employed the values Δ_0min⁡_ = 3.9 mK and Δ_0max⁡_ = 10 K extracted from fitting the pure SiO_2_ data of Figure 7 (inset).

Glass type	*x*	*A*_2LS_	*γ*	*A*_ATS_	*D*_min⁡_	Γ
	mol	10^−5^	10^−8^ sJ^3^	10^−5^	K	10^−6^ sK^5^
SiO_2_	0	47.2	5.30	—	—	—
AlBaSiO	—	116.2	13.40	264.7	0.65	69.73
K-Si	0.05	104.1	1.33	75.5	0.87	3.55
K-Si	0.08	146.5	1.23	130.0	0.87	3.97
K-Si	0.10	158.5	1.15	160.0	0.87	5.08
K-Si	0.20	239.5	0.82	281.9	0.87	6.44

**Table 2:** Extracted parameters for fits to the heat capacity data for SiO_2_ and (SiO_2_)_1−*x*_(K_2_O)_*x*_ glasses, with *D*_min⁡_ = 0.87 K and *V*_0_ = −0.42 K as fixed.

Glass type	*x*	*B*_ph_ × 10^8^	*B*_2LS_ × 10^8^	*B*_ATS_ × 10^8^	*x*_*j*_
	mol	Jm^−3^ K^−4^	Jm^−3^ K^−2^	Jm^−3^ K^−4^	ppm
SiO_2_	0	245.55	70.65	—	—
K-Si	0.05	260.92	155.23	22.77	29.86
K-Si	0.08	266.36	196.11	36.44	18.15
K-Si	0.10	269.46	221.62	45.55	10.54
K-Si	0.20	281.42	337.19	91.11	3.00

## Appendix

We first show how the spectrum of the 4LS Hamiltonian, (4), is similar to that of the 3LS Hamiltonian, (3) in the near-degenerate limit. We rewrite the *H*_0_^(4)^ 4LS Hamiltonian in the presence of a magnetic field, coupled orbitally to the charged tunneling particle:

(A.1) $$\begin{array}{c} H_{0}^{\left(4\right)}=\left(\begin{array}{cccc} E_{1} & D_{1}e^{i\phi /4} & D_{2}e^{i\phi /2} & D_{1}e^{-i\phi /4}\\ D_{1}e^{-i\phi /4} & E_{2} & D_{1}e^{i\phi /4} & D_{2}e^{i\phi /4}\\ D_{2}e^{-i\phi /4} & D_{1}e^{-i\phi /4} & E_{3} & D_{1}e^{i\phi /4}\\ D_{1}e^{i\phi /4} & D_{2}e^{-i\phi /2} & D_{1}e^{-i\phi /4} & E_{4} \end{array}\right), \end{array}$$

where ∑_*i*=1_^4^ *E*_*i*_ = 0 is imposed, *D*_1_ and *D*_2_ are the n.n., and n.n.n. hopping energies, respectively, and where

(A.2) $$\begin{array}{c} \phi =2\pi \frac{\Phi \left(B\right)}{\Phi_{0}},\;\Phi \left(B\right)=S_{⋄}\cdot B,\;\;\Phi_{0}=\frac{hc}{q}, \end{array}$$

is the Aharonov-Bohm phase resulting from the magnetic flux Φ(**B**) threading the square-loop (having area *S*_⋄_) closed trajectory of the particle. The above Hamiltonian should in fact be symmetrised over its permutations, since the sign of the n.n.n. Peierls phase is ambiguous (in practice, one replaces *D*_2_ *e* ^±*iϕ*/2^ with *D*_2_cos⁡(*ϕ*/2) in the appropriate matrix entries). The eigenvalues equation giving the energy levels is then as follows:

(A.3) ℰ4+ℰ2(∑i<jEiEj−4D12−D22(1+cos⁡ϕ))  −ℰ(∑i<j<kEiEjEk+4D12D2(1+cos⁡ϕ))  +E1E2E3E4−D12(E1E2+E2E3+E3E4+E4E1)  −12D22(E1E3+E2E4)(1+cos⁡ϕ)−2D12D22(1+cos⁡ϕ)  +2D14(1−cos⁡ϕ)+18D24(3+4cos⁡ϕ+cos⁡⁡(2ϕ)) =0.

More instructive than numerically extracting the four exact roots *ℰ*_0,1,2,3_ (with *ℰ*_0_ < *ℰ*_1_ < *ℰ*_2_ < *ℰ*_3_) is for us the physically interesting limit case in which |*E*_*i*_/*D*_1_ | ≪1 and |*D*_2_/*D*_1_ | ≪1 (near-degeneracy of the four-welled potential). The above eigenvalue equation then becomes much easier to study:

(A.4) ℰ4+ℰ2(∑i<jEiEj−4D12)  −D12(E1E2+E2E3+E3E4+E1E4)  +2D14(1−cos⁡ϕ)≈0,

this being the eigenvalue equation of the reduced 4LS Hamiltonian

(A.5) $$\begin{array}{c} H_{0\;\text{red}}^{\left(4\right)}=\left(\begin{array}{cccc} E_{1} & D_{1}e^{i\phi /4} & 0 & D_{1}e^{-i\phi /4}\\ D_{1}e^{-i\phi /4} & E_{2} & D_{1}e^{i\phi /4} & 0\\ 0 & D_{1}e^{-i\phi /4} & E_{3} & D_{1}e^{i\phi /4}\\ D_{1}e^{i\phi /4} & 0 & D_{1}e^{-i\phi /4} & E_{4} \end{array}\right), \end{array}$$

always for |*E*_*i*_/*D*_1_ | ≪1, and which has the following solutions:

(A.6) ℰ0,1,2,3D1=±12{4−∑i<jEiEjD12±[(4−∑i<jEiEjD12)2+4E1E2+E2E3+E3E4+E4E1D12+8(cos⁡ϕ−1)]1/2}1/2.

The perhaps surprising result is an energy spectrum where only the middle doublet (*ℰ*_1_, *ℰ*_2_ in our notation) becomes near-degenerate at weak (or zero) magnetic fields (*ϕ* → 0). This is shown in the inset of Figure 14 and is reminiscent of the situation with dimerized 2LS considered in [67] in order to account for the oscillations of the dielectric constant with *B*. Beside there being no evidence for a dimerization of TS in glasses (unlike perhaps in mixed and disordered crystals), one would have to explain why the ground state *ℰ*_0_ is prohibited for the tunneling particle (the real energy gap being in fact Δ*ℰ* = *ℰ*_1_ − *ℰ*_0_). The way out can be found again in Sussmann's paper [45] since the *n*_*w*_ = 4 welled trapping potential giving rise to the same physics as our 3LS must in fact have tetrahedral and not square geometry. The tetrahedral 4LS in a magnetic field will be considered elsewhere [59]. Here we only want to remark that the tetrahedral situation can be mimicked by a square multiwelled potential in which |*D*_1_/*D*_2_ | ≪1 and always in the limit case |*E*_*i*_/*D*_2_ | ≪1. This corresponds to the counterintuitive situation in which it is easier for the particle to tunnel across the square to the n.n.n. site rather than to a n.n. site, as if the middle potential barrier had collapsed. In this limit case, (A.4) becomes, instead,

(A.7) ℰ4+ℰ2(∑i<jEiEj−2D22cos⁡2ϕ2)  −D22(E1E3+E2E4)+D24cos⁡4ϕ2≈0,

with, once more, easily found solutions

(A.8) ℰ0,1,2,3D2=±12{2cos⁡2ϕ2−∑i<jEiEjD22±[(2cos⁡2ϕ2−∑i<jEiEjD22)2+4E1E3+E2E4D22−4cos⁡4ϕ2]1/2}1/2,

as exemplified in Figure 14 (main). We, therefore, obtain that the lowest-lying gap remains near-degenerate for weak fields, and we can conclude, therefore, that the lowest-lying eigenvalues display, at low-*T*, almost the same physics as in the case of a 3LS (Figure 5). This shows the important role of the frozen solid surrounding the tunneling “particle” (which could be, perhaps, a vacuum in fact) and that when nested within an incipient crystallite a magnetic-field-sensitive TS is well described by a tunneling 3LS as the minimal generic model potential.

Next, we seek a description of a cluster of *N*-correlated tunneling particles (atoms, ions, or molecules) and derive the transformation rules for the tunneling parameters (including also those involved in the theory for the magnetic effects [21]) when the cluster is replaced by a single “tunneling particle” as the result of the coherent tunneling (CT) of the particles in the cluster.

The “tunneling particle” in question is only a fictitious one, as was inferred in Section 2 by examining local minima in the energy landscape, representing the CT of a cluster of *N* true tunneling particles (which in the real glasses might be the lighter species involved in the material: Li^+^ in the disordered crystal Li : KCl, O^2−^ in the multisilicates, and H^+^ and/or D^+^ in *a*-glycerol) and for which we have to make up appropriate renormalized tunneling parameters. The concept of CT in separate local potentials is distinct from that of the joint tunneling of *N* particles in the same local potential, for in the latter case the tunneling probability would be depressed exponentially: $D_{0}/\hbar \approx \Omega {\left(\Delta_{0}/\hbar \Omega \right)}^{\sqrt{N}}$ (Δ_0_ being the real particles' common tunneling transparency). As we shall show below, at least for moderate values of *N*, for CT in separate potentials we expect instead:

(A.9) $$\begin{array}{c} D_{0}\approx N\Delta_{0},\;\;D_{\min}\approx N\Delta_{\min}, \end{array}$$

and, for the fictitious particle's charge and flux-threaded area (see [21] for the magnetic effects):

(A.10) $$\begin{array}{c} q=Nq_{0},\;\;S_{\Delta }\approx 4Na_{0}^{2}, \end{array}$$

where *q*_0_ = *O*(*e*) is the charge of the real tunneling particles and *a*_0_ Bohr's radius). In the latter relations, less obvious is the renormalization of the flux-threaded area *S*_Δ_ of a 3LS ATS. It is however the direct consequence of our multiphase model of a real glass, thought of as made up of regions of enhanced atomic ordering (RER) or microcrystals (Figures 3 and 6) embedded in a homogeneously disordered host matrix. The magnetic flux appears quadratically in our theory [21], each elementary flux adding up within each microcrystallite or RER and then appearing, squared, multiplied by cos⁡^2^ *β* in the glassy matrix in a magnetic field (*β* being the random angle formed by **S**_Δ_ with the magnetic field **B**), a factor averaging out to 1/2 in the bulk. From these considerations and from (A.9) and (A.10), the renormalization of the composite phenomenological parameter *D*_0_ | *q*/*e* | *S*_Δ_ would be as follows (if *q* = 2*e*, appropriate for the multisilicates):

(A.11) $$\begin{array}{c} D_{0}\left|\frac{q}{e}\right|S_{\Delta }\approx 8N^{3}\Delta_{0}a_{0}^{2}. \end{array}$$

Setting Δ_0_ = 1 mK, one gets a value of *N* ranging from about 25 coherent-tunneling particles in a cluster at the lowest temperatures [59], to about 600 at the higher temperatures. These estimates are somewhat speculative, since the real values of the elementary flux-threaded area and of the elementary tunneling barrier transparency Δ_0_ are unknown, we are however inclined to support the value *N*≈ 200 that was proposed by Lubchenko and Wolynes [29]. This would yield a value of Δ_min⁡_ ranging from 80 *μ*K to 4 mK also for the mixed alkali-silicate glasses (for which *D*_min⁡_ ≈ 800 mK). The above considerations show all in all the tendency for the coherent-tunneling cluster size *N* to be also temperature dependent.

We now come to the justification of (A.9). At low temperatures, the interactions between true tunneling particles become important and coherent-tunneling motion can take place. Coherent motion in the context of the tunneling model is a state in which all of the particles in each local potential contribute to the overall tunneling process in a correlated way. We exemplify our ideas in the context of the simplest 2LS situation first. Let us consider two interacting 2LS. Let the positions of the particles in the two wells be left (*L*) and right (*R*). The tunneling particles in the cluster interact via a weak potential *U* which may have its origin, for example, from either a strain-strain interaction having the form *U* ~ *A*/*r* ^3^ (dipole-dipole interaction) [29, 68], where *r* is the distance between a pair of tunneling particles either in the *L* or *R* well and *A* is a constant, or it could be due to electrostatic dipole-dipole interaction. The tunneling of the particle in one 2LS from *L* to *R* (or vice versa) influences, via the interaction, the particle in the other 2LS, forcing it to jump into the free well. The hopping Hamiltonian of two interacting 2LS can be written as follows (with Δ_*iL*_ = −Δ_*iR*_ = Δ_*i*_ and dropping factors of −1/2):

(A.12) H2=∑a=L,RΔ1ac1a†c1a+Δ01∑a≠a′c1a†c1a′+hc+∑aΔ2ac2a†c2a+Δ02∑a≠a′c2a†c2a′  +hc−U(c1L†c1Lc2L†c2L+c1R†c1Rc2R†c2R),

which favors coherent *LL* → *RR* and *RR* → *LL* joint tunneling and acts on the joint states |*aa*′〉 = |*LL*〉, |*LR*〉, |*RL*〉, |*RR*〉. The coherent motion of the two real particles can now be replaced by the tunneling of a new, fictitious particle in its own double well. In order to write the renormalized Hamiltonian of two coherent-tunneling particles, we are interested only in the matrix elements 〈*LL* | *H*_2_ | *LL*〉, 〈*RR* | *H*_2_ | *RR*〉, 〈*RR*|*H*_2_|*LL*〉, and 〈*LL* | *H*_2_ | *RR*〉 of Hamiltonian (A.12):

(A.13) $$\begin{array}{c} \langle LL\left|H_{2}\right|LL\rangle =\Delta_{1}+\Delta_{2}-U\\ \langle RR\left|H_{2}\right|RR\rangle =-\Delta_{1}-\Delta_{2}-U\\ \langle RR\left|H_{2}\right|LL\rangle =\langle LL\left|H_{2}\right|RR\rangle =\Delta_{01}+\Delta_{02}; \end{array}$$

instead of the latter two, the pair 〈*RL* | *H*_2_ | *LR*〉 and 〈*LR* | *H*_2_ | *RL*〉, having the very same value Δ_01_ + Δ_02_, could have served the purpose. These matrix elements represent the Hamiltonian of the fictitious particle, which corresponds to both real particles tunneling coherently together:

(A.14) $$\begin{array}{c} H_{1}^{\prime }=\left(\begin{array}{cc} \Delta_{1}+\Delta_{2}-U & \Delta_{01}+\Delta_{02}\\ \Delta_{01}+\Delta_{02} & -\Delta_{1}-\Delta_{2}-U \end{array}\right). \end{array}$$

The condition Δ_1_′ + Δ_2_′ = 0 is to be fixed through the addition of an overall constant. Next, we consider the case of three interacting 2LS and repeat the previous considerations. The Hamiltonian of three interacting 2LS has the form:

(A.15) H3=∑i=13{∑a=L,RΔiacia†cia+∑a≠a′(Δ0icia†cia′+hc)}−U∑i<i′ ∑acia†ciaci′a†ci′a.

The matrix elements of a single replacing fictitious particle that correspond to CT are obtained as follows:

(A.16) $$\begin{array}{c} \langle LLL\left|H_{3}\right|LLL\rangle =\Delta_{1}+\Delta_{2}+\Delta_{3}-3U\\ \langle RRR\left|H_{3}\right|RRR\rangle =-\Delta_{1}-\Delta_{2}-\Delta_{3}-3U\\ \langle RRR\left|H_{3}\right|LLL\rangle =\langle LLL\left|H_{3}\right|RRR\rangle =\Delta_{01}+\Delta_{02}+\Delta_{03}, \end{array}$$

(the choice of the latter two not excluding the remaining coherent matrix elements pairs: 〈*RR* *L* | *H*_3_ | *LL* *R*〉 and 〈*LL* *R* | *H*_3_ | *RR* *L*〉, 〈*R* *LR* | *H*_3_ | *LR* *L*〉 and 〈*LR* *L* | *H*_3_ | *R* *LR*〉, and 〈*L* *RR* | *H*_3_ | *R* *LL*〉 and 〈*R* *LL* | *H*_3_ | *L* *RR*〉, which are all equivalent). One can notice that the renormalized tunneling parameter is the sum of the Δ_0*i*_ of each 2LS. The energy asymmetry is also the arithmetic sum of the Δ_*i*_ of each 2LS, but one must add the interaction energy −*U* multiplied by *N*(*N* − 1)/2. Thus, for a coherently tunneling cluster of *N* 2LS we find that the diagonal matrix element becomes, generalizing to arbitrary *N*: Δ = ∑_*i*=1_^*N*^Δ_*i*_ − (*N*(*N* − 1)/2)*U* and the off-diagonal element, that corresponds to the CT-splitting for all *N* particles, becomes simply Δ_0_ = ∑_*i*_Δ_0*i*_.

Applying the previous considerations to our model for a number *N* of ATS with three wells (see Figure 15), we can write the interacting Hamiltonian in the form:

(A.17) HN=∑i=1N{∑a=13Eiacia†cia+∑a≠a′(D0icia†cia′+hc)}−U∑i<i′ ∑acia†ciaci′a†ci′a.

If we represent the group of *N* coherently tunneling particles as a single fictitious particle moving in a three-welled potential, which is characterized by its own ground-state energies *E*_*A*_ and tunneling parameter *D*_0_, we can describe this renormalized 3LS by the following Hamiltonian:

(A.18) $$\begin{array}{c} H_{1}^{\prime }=\sum_{A=1}^{3}E_{A}c_{A}^{†}c_{A}+\sum_{A\neq A^{\prime }}D_{0}c_{A}^{†}c_{A^{\prime }}+\text{hc}. \end{array}$$

The ground-state energies *E*_*A*_ in the wells and tunneling parameter *D*_0_ for the fictitious particle, in line with the calculations above, can be obtained through

(A.19) EA=〈aa⋯a|HN|aa⋯a〉 A=a=1,2,3D0=〈a′a′⋯a′|HN|aa⋯a〉=〈aa⋯a|HN|a′a′⋯a′〉 a≠a′,

(and the remaining variants of the second definition line, all equivalent). In analogy with the 2LS considerations, one can see that the renormalized tunneling parameters $D=\sqrt{E_{1}^{2}+E_{2}^{2}+E_{3}^{2}}$ and especially *D*_0_ can be replaced by the arithmetic sums of those of the bare coherently tunneling particles, *D* ≈ *ND*_*i*_ (neglecting the correction for a sufficiently weak *U* and moderate values of *N*) and *D*_0_ ≈ *ND*_0*i*_, respectively. Indeed, the tunneling probabilities of weakly correlated events should add up for values of *N* not too large. Therefore, since *N* can attain values as large as 200 [29] (independently of the solid's composition) in some models, and as corroborated by our reasoning in this appendix, this leads to values of *D*_*i*_ and *D*_0*i*_ (as extracted from our theory's fitting parameters) comparable to those characteristic of the 2LS-TM. The large values of *D*_min⁡_ and especially of *D*_0min⁡_ and *D*_0max⁡_, as extracted from our fits of our theory to the available experimental data, find therefore an interesting and physically plausible explanation.
